# The Role of Smoothened-Dependent and -Independent Hedgehog Signaling Pathway in Tumorigenesis

**DOI:** 10.3390/biomedicines9091188

**Published:** 2021-09-10

**Authors:** Jian Yi Chai, Vaisnevee Sugumar, Mohammed Abdullah Alshawsh, Won Fen Wong, Aditya Arya, Pei Pei Chong, Chung Yeng Looi

**Affiliations:** 1 School of Biosciences, Faculty of Health & Medical Sciences, Taylor’s University, 1 Jalan Taylors, Subang Jaya 47500, Malaysia; jianyi.chai@sd.taylors.edu.my (J.Y.C.); PeiPei.Chong@taylors.edu.my (P.P.C.); 2 School of Medicine, Faculty of Health & Medical Sciences, Taylor’s University, 1 Jalan Taylors, Subang Jaya 47500, Malaysia; vaisneveesugumar@sd.taylors.edu.my; 3 Department of Pharmacology, Faculty of Medicine, University of Malaya, Kuala Lumpur 50603, Malaysia; 4 Department of Medical Microbiology, Faculty of Medicine, University of Malaya, Kuala Lumpur 50603, Malaysia; wonfen@um.edu.my; 5 School of Biosciences, Faculty of Science, Building 184, The University of Melbourne, Melbourne, VIC 3010, Australia; aditya.arya@unimelb.edu.au; 6 Centre for Drug Discovery and Molecular Pharmacology (CDDMP), Faculty of Health & Medical Sciences, Taylor’s University, 1 Jalan Taylors, Subang Jaya 47500, Malaysia

**Keywords:** GLI1 protein, hedgehog pathway, mutations, epigenetic regulation, glioma-associated oncogene, noncanonical, cancer, clinical trial, hedgehog inhibitors

## Abstract

The Hedgehog (Hh)-glioma-associated oncogene homolog (GLI) signaling pathway is highly conserved among mammals, with crucial roles in regulating embryonic development as well as in cancer initiation and progression. The GLI transcription factors (GLI1, GLI2, and GLI3) are effectors of the Hh pathway and are regulated via Smoothened (SMO)-dependent and SMO-independent mechanisms. The SMO-dependent route involves the common Hh-PTCH-SMO axis, and mutations or transcriptional and epigenetic dysregulation at these levels lead to the constitutive activation of GLI transcription factors. Conversely, the SMO-independent route involves the SMO bypass regulation of GLI transcription factors by external signaling pathways and their interacting proteins or by epigenetic and transcriptional regulation of GLI transcription factors expression. Both routes of GLI activation, when dysregulated, have been heavily implicated in tumorigenesis of many known cancers, making them important targets for cancer treatment. Hence, this review describes the various SMO-dependent and SMO-independent routes of GLI regulation in the tumorigenesis of multiple cancers in order to provide a holistic view of the paradigms of hedgehog signaling networks involving GLI regulation. An in-depth understanding of the complex interplay between GLI and various signaling elements could help inspire new therapeutic breakthroughs for the treatment of Hh-GLI-dependent cancers in the future. Lastly, we have presented an up-to-date summary of the latest findings concerning the use of Hh inhibitors in clinical developmental studies and discussed the challenges, perspectives, and possible directions regarding the use of SMO/GLI inhibitors in clinical settings.

## 1. Introduction

The hedgehog (Hh) signaling pathway was first discovered in *Drosophila melanogaster’s* embryonic cell [1]. Its evolution across various species is relatively conserved; however, duplication of the Hh gene in mammals revealed the involvement of three other members of the Hh family, namely the Sonic (Shh), Desert (Dhh), and Indian hedgehog (Ihh) [2]. Each of these genes has a diverse function in developing various tissues and organs; nevertheless, they utilize a similar pathway to be activated. In mammals, the Hh signaling is a rather complex relay mechanism that occurs in the primary cilium. Without the binding of Hh ligands to the Patched (PTCH), a 12-pass transmembrane protein receptor, the pathway remains suppressed due to the inhibitory effect of PTCH on the seven-pass transmembrane protein Smoothened (SMO) [3]. The binding of Hh ligands to PTCH relieves the inhibition of SMO protein, allowing its translocation into the primary cilium, where it rapidly accumulates [4]. Subsequently, activated SMO interferes with the proteolytic processing of glioma-associated oncogene homolog (GLI) proteins and promotes their dissociation from Suppressor of Fused (SUFU), allowing their translocation into the nucleus [5]. Through their DNA-binding domains, GLI activators (GLIAs) then bind to the GLI-binding consensus sequence 5′-GACCACCCA-3′ residing within promoters of target genes to initiate their gene transcription, such as cyclins (*CCND1*, *CCND2*), antiapoptotic factors (*BCL2*, *BCLX*), migratory genes (*SNAI1*, *ZEB1*), and its own pathway genes (*PTCH1*, *GLI1*) [6,7].

**Figure 1:**
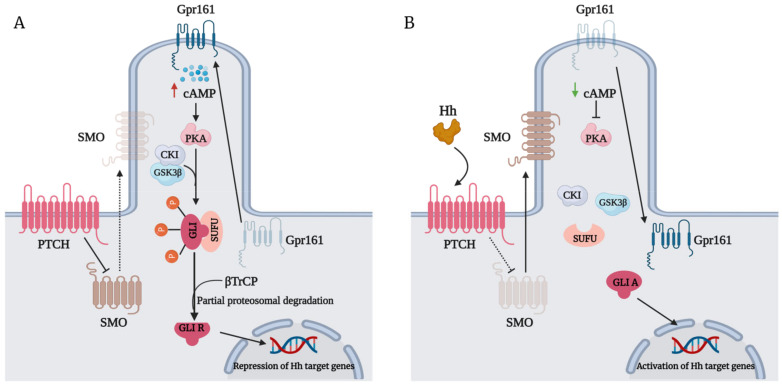
(A) The repression of Smoothened (SMO) by the Patched (PTCH) receptor in the absence of hedgehog (Hh) ligands promotes the interaction of Suppressor of Fused (SUFU) and glioma-associated oncogene homolog (GLI). G-protein coupled receptor 61 (GPR161) translocates to the primary cilium, which triggers high levels of cyclic adenosine monophosphate (CAMP). Elevated ciliary levels of CAMP maintain high levels of protein kinase A (PKA) activity, which phosphorylate GLI at P1-6 clusters. Consequently, phosphorylation of GLI by PKA prime its phosphorylation by casein kinase I (CKI) and glycogen synthase kinase 3 beta (GSK3β) further. Phosphorylated GLI is recognized by the β-TrCP, promoting its ubiquitination and partial proteasomal processing into a repressor. GLI repressor (GLIR) then translocates into the nucleus to repress target gene transcription. (**B**) The binding of the Hh ligand to the PTCH receptor alleviates its repression of SMO, allowing SMO translocation to the primary cilium. Activated SMO inhibits SUFU, allowing the dissociation of GLI from SUFU. Additionally, Gpr161 is removed from the primary cilium, causing low CAMP levels and PKA activity. The release of GLI from SUFU and low PKA activity results in the dephosphorylation of GLI, preventing its proteasomal processing into a repressor. Full-length GLI or GLI activator (GLIA) then translocates into the nucleus to transcribe target genes. Red upward triangle-headed arrow: upregulation; green downward triangle-headed arrow: downregulation; dotted black triangle-headed arrow: inactivation; bar-headed arrow: inhibition; dotted bar-headed arrow: loss of inhibition.

However, the diverse response of GLI in tissues is very dependent on the delicate balance between GLIAs and GLI repressors (GLIRs) combined. The negative regulation of GLI protein (Figure 1A) is regulated by its interaction with SUFU by virtue of its SUFU-binding domain. In the absence of the Hh ligand, SMO remains inactivated, which allows the tight association of SUFU with GLI [8]. GLI bound to SUFU is susceptible to phosphorylation events that promote its processing into repressors. G-protein coupled receptor 61 (Gpr161) localizes to the primary cilia to maintain high cyclic adenosine monophosphate (CAMP) levels and protein kinase A (PKA) activity [9], which phosphorylate P1-6 clusters located on GLI2/3 [10]. Their phosphorylation by PKA primes their subsequent phosphorylation by glycogen synthase kinase 3 beta (GSK3β) and casein kinase I (CKI) [11]. Phosphorylated GLI2/3 are recognized by the Cul1/β-TrCP complex, promoting their ubiquitination and subsequent proteasomal-dependent processing into GLIRs [12,13]. GLIRs then bind to the promoters of target genes to repress their transcription. In the presence of an Hh ligand, however, activation of SMO leads to the dephosphorylation of GLI2/3 P1-6 clusters and their dissociation from SUFU [10], favoring the translocation of GLIAs into the nucleus to initiate the transcription of target genes (Figure 1B). The expression of *GLI1*, a primary Hh target gene, serves to amplify Hh transduction at the transcriptional level further [14].

Typically, Hh signaling activation is classified into two general models: ligand-independent (Type I) and ligand-dependent (Type II and III) Hh signaling. This model centers around the various Hh pathway components leading to GLI activation, which can occur either through mutations in *PTCH* or *SMO* (ligand independent) or Hh ligand stimulation (ligand dependent); however, the transcriptional or epigenetic dysregulation of Hh pathway-related genes (e.g., aberrant methylation or excessive transcription factor activation) leading to GLI activation and the regulation of GLI beyond SMO transduction are often overlooked in this model. In this review, we describe a Hh signaling model that focuses on two different GLI regulation levels relevant to carcinogenesis: SMO-dependent and SMO-independent GLI activation. Arguably, these models provide a holistic view of the paradigms of hedgehog signaling networks involving GLI regulation at the SMO level or beyond and may be more relevant to current therapeutic strategies involving the development of SMO and GLI inhibitors for treating Hh-dependent cancers. Additionally, we present the latest clinical trial findings for the recent development of Hh inhibitors in cancer treatment and provide a comprehensive review concerning the relevance, limitations, and future perspective of SMO/GLI inhibitors as targeted cancer therapy. Importantly, GLI inhibitors have shown superior anticancer activity compared to inhibitors targeting upstream (Hh and SMO) of GLI in preclinical studies [15,16]. Furthermore, GLI inhibitors effectively suppress cancer growth in many GLI-dependent cancers that utilize an SMO-independent route of GLI regulation, of which treatment with upstream inhibitors has proven ineffective [17]. Thus, understanding GLI regulation paradigms is fundamental to developing novel GLI inhibitors worthy of moving forward to clinical settings, which may help set a new stage for Hh therapy in the future.

## 2. GLI Proteins and Their Domains

**Figure 2:**
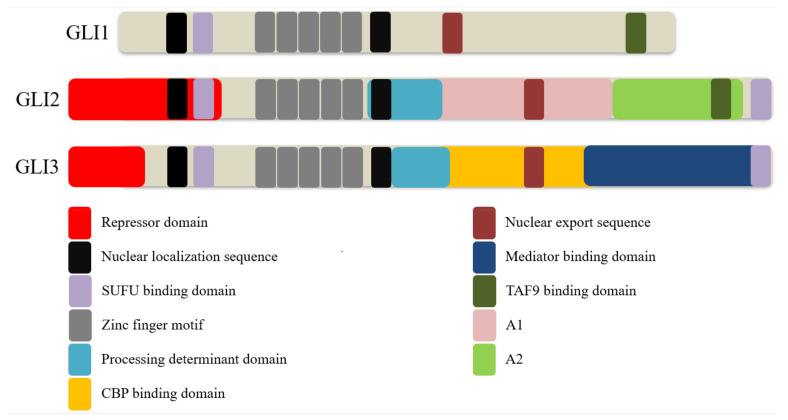
Schematic representation of the domains and motifs in glioma-associated oncogene homolog (GLI) proteins. All GLI proteins contain a well-conserved Supressor of Fused (SUFU)-binding domain, zinc finger motifs, nuclear localization sequences, and a nuclear export sequence. GLI2 and GLI3 contain both an N-terminal repressor and several C-terminal transactivation domains, unlike GLI1, which contains only a single transactivation domain reported so far. Additionally, GLI2 and GLI3 contain a second SUFU-binding domain at the C-terminal end critical for regulating nuclear GLI function. Both GLI2 and GLI3 contain a processing determinant domain that contributes to the proteolytic processing of these proteins into their repressor form with a more active role in GLI3 than GLI2. GLI2 contains two major transactivation domains, termed A1 and A2, while the GLI3 transactivation domain includes a CREB-binding protein (CBP)-binding domain and mediator-binding domain. Both GLI1 and GLI2 contain an α-helical herpes simplex viral protein 16-like activation domain that binds to TATA-box binding protein associated factor 9 (TAF9) due to the presence of a highly conserved FXXΦΦ (F = phenylalanine; X = any residue; Φ = any hydrophobic residue) motif in the domain. The FXXΦΦ motif is also conserved in GLI3 but does not bind to TAF9.

GLI is a part of the GLI-Kruppel family, characterized by the presence of C2H2-Kruppel-type zinc-finger (ZF) motifs [18]. Three homologs exist in vertebrates, namely GLI1, GLI2, and GLI3 (Figure 2). These proteins consist of overlapping domains, including a repressor and transactivation domain, and possess distinct but partially redundant functions. Since GLI1 lacks the repressor domain, it acts as a sole transcriptional enhancer. By contrast, GLI2 protein possesses both a repressor and two transactivation domains (TADs), A1 and A2, and acts as both a repressor and an activator. However, GLI2 mostly behaves as a transcriptional activator due to the inefficient processing of GLI2 into GLI2 repressor (GLI2R) [19]. Likewise, GLI3 protein possesses both repressor and activation domains but serves primarily as a transcriptional repressor due to an active processing determinant domain (PDD) that allows efficient processing of GLI3 into GLI3 repressor (GLI3R) [19,20]. Of note, GLI2 also contains a PDD but is inefficiently processed due to differences in amino residues critical for preventing complete degradation by the proteasome [20].

All three GLI homologs contain five Krüppel-like ZF motifs that recognize and bind to a nine base-pair DNA consensus motif 5′-GACCACCCA-3′. ZF4 and ZN5 mediate the binding of GLI proteins to the conserved DNA motif, while ZNF1-3 stabilizes the DNA domain through interaction with the phosphate backbone [21,22]. All three GLI homologs also contained a highly conserved SUFU-binding domain and two putative nuclear localization sequences (NLSs), including NLS1 and NLS2. NSL2 is a canonical bipartite NLS containing two basic clusters mapped to the fifth ZF motif in Ci and C-terminal side of GLI proteins, while NLS1 has features of both a canonical monopartite NLS and a noncanonical proline-tyrosine (PY)-NLS located just upstream of the SUFU-binding domain [23]. Both NLSs play a cooperative functional role in regulating the nuclear localization of GLI proteins, likely through importin (Imp)-α/β1 mediated nuclear import classic of canonical NLSs [24]. 

Mutations in either the NLS1 or NLS2 have been shown to partially impair the nuclear transportation of both GLI1 and GLI2, while the loss of both NLSs results in a drastic increase in cytoplasmic localization of both GLI proteins [25,26]. The function of NLS2 is also heavily regulated by a Thr374 residue adjacent to the first basic cluster of the bipartite motif. Phosphorylation of Thr374 residue by PKA enhanced the local negative charge nearby the NLS2, thus inhibiting NLS2 function and consequently inhibiting GLI1 nuclear accumulation [27]. The noncanonical PY-NLS feature of NLS1 has also been shown to mediate GLI2 and GLI3 nuclear transport by cooperating with karyopherin-β2 (Kapβ2) known to recognize PY-NLSs [28]. Besides regulating nuclear transportation, the interaction of PY-NLS and Kapβ2 also plays a major role in regulating the ciliary localization of all three GLI proteins independently of the Imp-α/β1 nuclear import system [25,29].

A leucine-rich nuclear export sequence (NES) is also found in all three mammalian GLI proteins, and their functional coordination with NLSs plays a major role in regulating the nuclear-cytoplastic shuttling of GLI proteins. The nuclear export of GLI1 and GLI2 is chromosomal region maintenance 1 (CRM1)-dependent, and inactivation of CRM1 with Leptomycin B (LMB) or the substitution of conserved leucine residues within the GLI1/GLI2 NES was shown to enhance the nuclear accumulation and transcriptional activities of both GLI proteins [26,30]. Interestingly, the shuttling of the SUFU-GLI1 complex between the cytoplasmic and nuclear compartments was found to depend on functional NES and CRM1, whereby the loss of either function led to an impaired cytoplasmic sequestration of GLI1 by SUFU and enhanced colocalization of SUFU and GLI1 in the nucleus [26].

SUFU binds to SUFU-binding domains located at N- and C-terminals of GLI proteins to regulate their activities through various mechanisms. Firstly, SUFU sequesters GLI proteins in the cytoplasm by binding to the SYGH core motif within the N-terminal domain of GLI proteins [31]. This interaction facilitates the phosphorylation of GLI proteins by PKA, GSK3β, and CK1, leading to their proteasomal degradations or processing into repressors [8,12]. Secondly, SUFU prevents the nuclear translocation of GLI proteins by masking their NLSs. The binding of SUFU to the SYGH core motif within the N-terminal domain of GLI proteins blocks the binding of Impβ1 and Kapβ2 to NLSs, which impedes the nuclear import of GLI proteins [24,28]. Thirdly, SUFU has been shown to regulate Ci/GLI transcriptional activity inside the nucleus. The binding of SUFU to a second conserved C-terminal SUFU-binding domain of Ci masked the *Drosophila* CREB-binding protein (CBP)-binding domain (dCBD) located near the SUFU-binding domain, impeding the recruitment of the transcriptional coactivator CBP. In turn, the loss of CBP recruitment inhibited the transcriptional activity of Ci; the C-terminal SUFU-binding site was also conserved in GLI2 and GLI3, and deletion of this site partially blocked the SUFU-mediated inhibition of GLI2 transcriptional activity [31], recapitulating the effect of SUFU-binding at the C-terminal SUFU-binding domain in the impediment of CBP recruitment and suppression of Ci transcriptional activity.

SUFU also negatively regulates the transcriptional activity of GLI1 in the nucleus despite the loss of CRM1-dependent nuclear export (inhibited by LMB), suggesting that the repressive activity of SUFU can still occur in the nucleus independent of cytoplasmic sequestration [26]. In further support of this finding, SUFU was also shown to interact directly with GLI1 bound to DNA, raising the possibility that this interaction may hinder the binding of other transcription activators with GLI1 [30]. By contrast, Zhang et al. argued that SUFU does not serve merely as a negative regulator but rather as a chaperone protein with a unifying role in regulating the function of GLI proteins [32]. Evidently, SUFU accompanied the translocation of GLI1 into and GLIR out of the nucleus. Furthermore, SUFU also accompanied GLI1 but not GLIR to the ciliary tip, a necessary step that precedes the translocation of GLI1 into the nucleus. Importantly, SUFU facilitates the binding of GLI1 to GLI-binding sites on the chromatin, while concomitantly reducing GLI3 binding, and intact SUFU expression is required for maximal Shh signaling output needed for the specification of the most ventral neurons [32]. 

The *Drosophila* CBP (dCBP) has been shown to bind to the dCBD of Ci as a coactivator, while the loss of dCBP abolished Hh signaling [33]. Sequence alignment revealed a motif fairly well conserved between the dCBP-binding domain of Ci and the A1 domain of GLI2 [34], but the role of CBP in GLI2 activity has yet to be elucidated. Like Ci, GLI3 also possesses a CBP-binding domain (CBD) and utilizes CBP as a coactivator for its transcriptional activity [35]. By contrast, Zhou et al. reported that CBD showed weak transactivation in vivo, but CBP could bind efficiently to the Mediator-binding domain (MBD) located upstream of CBD to promote GLI3 transactivation, suggesting a concerted functional interaction between CBP and RNA polymerase II transcriptional mediator complex [36]. Besides binding CBP, MBD also physically targeted and inhibited the MED12 interface in the mediator complex, which in turn reversed the mediator-dependent suppression of GLI3 transactivation activity [36]. By contrast, CBP does not bind to GLI1 [35], suggesting the lack of a CBD or MBD in GLI1.

The C-terminal end of the GLI1 contains an α-helical herpes simplex viral protein 16-like activation domain, including a highly conserved FXXΦΦ (F = phenylalanine; X = any residue; Φ = any hydrophobic residue) motif recognizing TAFII31/TATA-box binding protein associated factor 9 (TAF9) subunit of general transcription factor II D [37]. This motif is fairly conserved in the A2 domain of GLI2 and the C-terminal end of GLI3 [37,38]. However, TAF9 binds only to GLI1 and GLI2 but not GLI3 to promote their transcriptional activities, suggesting a redundancy of the FXXΦΦ motif in GLI3. Conversely, binding interference between GLI proteins and TAF9 by mutating the FXXΦΦ motif resulted in the loss of transcriptional activities of GLI proteins [37,38,39].

Both GLI2 and GLI3 contain an N-terminal repressor domain (RD) that exerts repressive transcriptional activity upon proteolytic removal of their C-terminal TADs. In contrast to the TAD of GLI proteins, their RDs are less well characterized in terms of their motifs and binding partners. The RD is most well defined for its interaction with the histone deacetylase (HDAC) complex. Ski was shown to interact directly with the N-terminal domain of both GLI3R and full-length GLI3 and to form a complex with HDAC1 to promote GLI3-mediated transcriptional repression. Additionally, a Ski-binding site was also mapped to the N-terminal RD of GLI2. Conversely, *Ski*-deficient mouse embryonic fibroblast (MEF) efficiently abrogated GLI3 and GLI2 transcriptional repressive activities. Ski forms complexes with corepressors such as N-CoR/SMRT, mSin3, and Sno to recruit HDACs necessary to mediate transcriptional repression activities of other repressors [40]. Mouse SUFU has been shown to interact with SAP18, a member of the mSin3-HDAC corepressor complex, to enhance GLI3-mediated transcriptional repression and impaired GLI1 transcriptional activity. Functionally, mouse SUFU interacted with GLI1, possibly via the SYGF motif in the N-terminal SUFU-binding domain and recruited the mSin3-HDAC complex through interaction with SAP18 to impede GLI1 transcriptional activity. It is conceivable that the same process may also occur in GLI3 to potentiate GLI3 transcriptional repressive activity, as both GLI1 and GLI3 interact with SUFU via the same SYGH motif at the N-terminal end. Furthermore, the Ski binding site overlaps the SUFU-binding domain at the N-terminal region of GLI3, suggesting a possible functional cooperative role between SUFU and Ski in recruiting the HDAC corepressor complex to promote GLI3-mediated transcriptional repression activity [41].

## 3. The Mechanism of GLI Regulation in Human Cancers

Aberrant GLI activation can occur via SMO-dependent or SMO-independent routes. SMO-dependent activation of GLI can result from two mechanisms: mutations that lead to the loss-of-function of the major negative regulator protein PTCH1 and gain-of-function of the SMO protein or dysregulated expression of the Hh/PTCH1/SMO caused by aberrant transcriptional and epigenetic regulations. This route of GLI activation includes both ligand-dependent and ligand-independent Hh signaling. On the other hand, the noncanonical SMO-independent activation of GLI can occur in the absence of Hh ligand binding to the PTCH receptor, as GLI activation is regulated by various other oncogenic pathways and signaling proteins external to the Hh pathway; this route of GLI activation is exclusively ligand independent. Accumulating evidence has implicated both routes of GLI regulation in the development of many known cancers. Because GLI plays such a crucial role in regulating developmental and cellular processes such as embryogenesis, differentiation, stem cell maintenance, and proliferation, it is understandable that its unregulated activation plays a big part in cancer tumorigenesis. Thus, this section highlights the SMO-dependent and SMO-independent mechanisms by which GLI is regulated to induce tumorigenesis.

Due to the vast amount of proto-oncogenes they regulated, GLI proteins are closely associated with alterations of cancer hallmarks, including sustained proliferative signals, evading growth suppressors, resisting cell death/apoptosis, avoiding immune destruction, activating migration/invasion and metastasis, genomic instability and mutations, tumor-promoting inflammation, and inducing angiogenesis [42]. For instance, the transcriptional upregulation of D-type cyclins, *CCND1* and *CCND2*, by GLI proteins facilitates the bypass of mitotic cellular checkmarks, leading to enhance cell cycling and uncontrolled proliferation [43]. In the presence of cytotoxic drugs, GLI proteins can transcriptionally upregulate the expression of *BCL2* or transporter proteins to inhibit the activation of apoptotic signaling cascades and promote drug efflux, thus resisting drug-induced cell death [44]. The upregulation of GLI proteins in cancers is also associated with the downregulation of p53, which impairs cell cycle arrest and enhances genetic instability [45]. GLI proteins upregulate the expression of invasion-related and mesenchymal proteins, such as matrix metalloproteinases, N-cadherin, vimentin, and SNAI1, to activate cancer migration, invasion, and metastasis [46].

**Figure 3:**
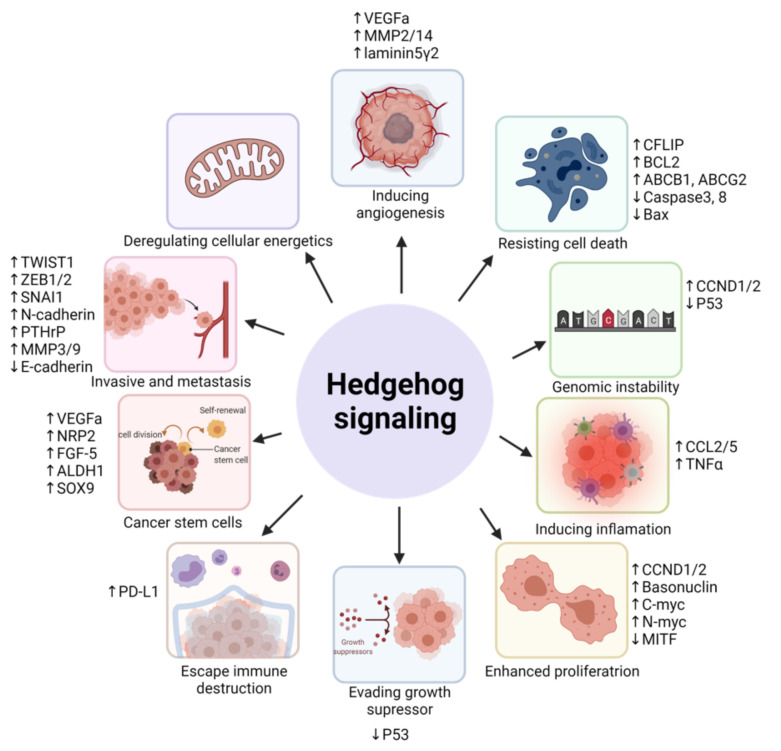
A simplified diagram on the role of Hh signaling activation in driving cancer hallmarks. Upward triangle-headed arrow: upregulation; downward triangle-headed arrow: downregulation.

A new but notable cancer hallmark involving the dedifferentiation of noncancer stem cells to stem cell- or tumor-initiating-like cells has also been proposed by Senga and Grose [47] and poses great relevance to Hh-GLI signaling. Evidently, activation of GLI proteins has been associated with the acquisition of cancer stem cell (CSC)-like traits through upregulation of genes involved in dedifferentiation, self-renewal, and pluripotency, leading to enhanced tumorigenicity and drug resistance [48]. Thus, understanding the complex regulatory network of GLI activation can assist the development of more than one therapeutic approach in overcoming the hallmarks of cancer. In the next part, we focused on the SMO-dependent and -independent mechanisms by which GLI is activated and their relevance to cancer hallmarks (Figure 3, Table 1).

### 3.1. SMO-Dependent GLI Activation

#### 3.1.1. Mutations of Hh Pathway Genes Upstream of GLI

**Figure 4:**
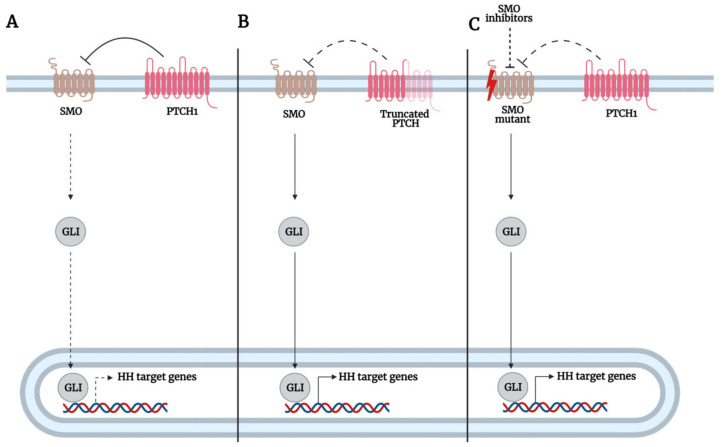
A simplified illustration of Smoothened (SMO)-dependent glioma-associated oncogene homolog (GLI) regulation in the context of hedgehog (Hh) pathway mutations. (**A**) Under physiological conditions in adult tissues, Patched1 (PTCH1) functions by inhibiting SMO, which represses GLI function and prevents its translocation into the nucleus to activate target genes transcriptionally. (**B**) However, in cancer cells, loss of heterozygosity (LOH) of *PTCH1* alleles results in the formation of a nonfunctional truncated PTCH1 protein. Consequently, this results in the constitutive activation of SMO, which promotes GLI activation and its translocation into the nucleus to activate Hh target genes transcriptionally. (**C**) Alternatively, constitutively active SMO M1/M2 mutants resistant to PTCH1 inhibition or inhibition by SMO inhibitors promote the sustained activation of GLI and its subsequent translocation into the nucleus to transcriptionally activate Hh target genes. Bar-headed arrow: inhibition; dotted bar-headed arrow: loss of inhibition; triangle-headed arrow: activation; dotted triangle-headed arrow: inactivation.

SMO protein represents the key positive regulator of GLI function. However, the mutation or dysregulation of SMO or any of the components such as Hh, PTCH1 leading to its activation leads to the aberrant activation of GLI and consequently oncogenesis (Figure 4). The majority of mutations in Hh signaling components have been mostly reported in cases of nevoid basal cell carcinoma syndrome (NBCCS) or Gorlin syndrome and basal cell carcinoma (BCC). The majority of *PTCH1* mutations result in prematurely truncated PTCH1 proteins that are inactivated, thus promoting constitutive activation of the Hh pathway. In people with Gorlin syndrome, they carry an inactivating germline mutation of one allele of the *PTCH1* gene that predisposes them to multiple developmental abnormalities such as odontogenic keratocysts and skeletal anomalies. Consequently, when the second wild-type allele is inactivated, usually by somatic events such as UV or ionizing radiation exposures, it results in the loss of heterozygosity (LOH) of the *PTCH1* gene, which in turn promotes carcinogenesis in the form of multiple BCCs [129]. Similarly, in sporadic BCCs, inactivation of both alleles is required to kickstart the development of BCCs, but unlike hereditary BCCs, both alleles are inactivated as a result of somatic events [130]. As PTCH1 functions by repressing SMO, the loss of PTCH1 alleviates its repression of SMO and promotes the onset of GLI-mediated expression of Hh target genes and tumorigenesis. For instance, the loss of *PTCH1* in mutant skin resulted in BCC development in nude mice, associated with compromised epidermal differentiation and ectopic expression of Hh target genes, *GLI1* and *PTCH1*, in the interfollicular epidermis (IFE). Furthermore, the loss of PTCH1 in mutant skin resulted in aberrant expression of CCND1 and CCND2 and impaired expression of p53 tumor suppressor, resulting in increased cell cycling and impaired p53 response to cell-cycle-driven DNA damage, which enhanced genomic instability [45].

BCCs are also shown to overexpress GLI1 and GLI2 transcription factors, and oncogenic mutations in genes encoding these proteins are uncommon, suggesting that the mutation of *PTCH1* is sufficient to drive GLI1 activation and tumorigenesis. Evidently, transgenic mice overexpressing GLI1 developed tumors that closely resemble human BCCs. Additionally, GLI1-overexpressing transgenic mouse tumors showed differentiated marker expression similar to that of human BCCs [49]. *GLI2* gene silencing of nude mice injected with BCC-like K5-Gli2DN2 cell line resulted in a marked increase in apoptosis and decreased vascularization of BCC-like tumors that led to their retarded growth [50], as conditional GLI2 expression is required for the sustained growth of established BCCs [51]. In transgenic mice overexpressing *GLI2* driven by the keratin 5 (K5) promoter, CCND1 and CCND2 were significantly upregulated in BCC biopsies, promoting sustained tumor growth and expansion of BCC [62]. In human BCC tumors, GLI1 and GLI2 were shown to be significantly elevated biopsy tissues, and GLI1 and GLI2 transcriptionally upregulated *basonuclin* and *cFlip*, respectively, to promote cancer cell survival [44,52]. 

Basonuclin functions by enhancing the transcription of rRNA, a common feature in tumorigenesis. Notably, basonuclin expression was significantly higher in human BCC tissues than in unaffected epidermis. Moreover, BCC tissues expressing high levels of basonuclin were also elevated for their GLI1 expression. Mechanistically, GLI1 binds to GLI-binding sites within the *BNC1* (encoding for basonuclin protein) promoter to induce basonuclin transcription. Importantly, high levels of basonuclin correlated with elevated levels of 47S pre-rRNA expression, and both proteins were more common in infiltrative type BCC than nodular-type BCC. Moreover, elevated levels of 47S pre-rRNA were more common in a subset of BCC cells expressing high Ki67 cell cycle regulator levels, suggesting that basonuclin and 47S pre-rRNA may promote cell cycling and unrestricted growth of BCC. Notably, infiltrative-type BCCs are clinically more invasive and have greater growth potential as assessed by lesion size and proliferative Ki67 markers [131]. With regards to the above, GLI proteins could upregulate basonuclin and consequently enhance BCC cell proliferation by increasing rRNA transcription, which may lead to the development of a more aggressive subtype of BCC [52]. Indeed, Marceline et al. also reported that higher levels of PTCH1, which is among the main target genes of GLI, were more frequently detected in infiltrative rather than nodular-type BCC [132]

Immunohistochemical analysis of human BCC biopsies excised from different patients revealed that high expression of GLI2 was positively correlated with high expression of cFlip and BCL2. The silencing of GLI2 or cFlip was shown to increase the number of apoptotic cells induced by tumor necrosis factor-related apoptosis-inducing ligand (TRAIL) in BCC tissue ex vivo. Of note, cFlip functions as a master antiapoptotic regulator by inhibiting caspase 8 activation, a downstream target of TRAIL. Indeed, GLI2 expression in HaCaT keratinocytes cells was found to render them resistant to TRAIL-induced apoptosis by enhancing BCL2 expression and reducing caspase 8 activation [44]. Overall, the frequent loss of PTCH1 and the frequent activation of GLI in the absence of *GLI* mutations strongly suggest a role of SMO derepression by the loss of PTCH1 in mediating GLI activation in BCC.

To a smaller extent, mutations in *SMO* have also been reported in sporadic BCCs, leading to the Hh pathway’s constitutive activation [129,133]. Often, *SMO* mutations affecting ligand-binding pockets (LBPs) lead to the development of drug resistance toward SMO inhibitors. For instance, *SMO* missense mutation (G497W and D473Y) have been shown to contribute to primary and secondary resistance to vismodegib in BCC patients, respectively, by interfering with the binding of vismodegib to SMO LBP [63]. Interestingly, when treated with vismodegib, vismodegib-resistant tumors of BCC patients with *SMO* mutations (D473H, D473G, and W535L) had significantly higher levels of GLI1 compared to vismodegib-sensitive tumors. Moreover, inhibition of GLI function by GLI kinase atypical Protein Kinase C ι/λ (aPKC-ι/λ)/GLI inhibitor PSI and GLI2 inhibitor arsenic trioxide effectively suppressed Hh pathway activation in *Smo*^−/−^ MEFs expressing SMO with LBP mutations (D473G, W281C, H231R, and Q477E), suggesting that the SMO LBP mutant that constitutively promotes GLI expression and consequently Hh pathway activation in the presence of vismodegib can be circumvented with the use of GLI antagonists. Conversely, treatment of these cells with vismodegib or Shh-N (active fragment of Shh) did not affect Hh pathway activity [64]. A D473H SMO mutant was also found to confer resistance to vismodegib in a medulloblastoma patient and induced *GLI1* luciferase reporter activity in C3H10T1/2 cells [67]. Thus, as SMO mutants can constitutively activate Hh signaling by GLI activation to promote cancer cell survival, targeting GLI may serve as a promising second-line therapy for the treatment of SMO-inhibitor-resistant tumors.

The constitutively active SMOM2 mutant (W535L) was also found to be overexpressed in some sporadic and vismodegib-resistant BCCs. Notably, the SMOM2 mutant strongly induced Hh pathway activation in the absence of Hh ligand through GLI modulation and is able to resist the inhibitory catalytic signal of PTCH1. Furthermore, several other SMO mutants (F460, W535L, V321M, and L412F), including SMOM2, conferred resistance to vismodegib in *Smo*^−/−^ MEF cells, suggesting a dual role of SMO mutants in tumorigenesis by promoting a constitutive Hh pathway activation and endowing resistance [63]. 

To further support the oncogenic role of SMO mutants, transfection of embryonic fibroblast REF52 cells with SMOM1 (R562Q) and -M2 mutant was found to enhance *GLI1* transcript levels and confer cells’ ability to grow in soft agar. Furthermore, overexpression of the SMOM2 mutant in transgenic mice led to the development of abnormal skin features similar to BCC [65]. In the adult IFE cells, SMOM2 expression drove the formation of invasive-type BCC in transgenic mice, which was associated with enhanced expression of Hh pathway genes (*GLI1/2*, *PTCH1/2*, and *HHIP)* and embryonic hair follicle progenitor markers (P-cadherin, LHX2, and CUX1). The loss of PTCH1 in IFE cells also yielded similar results compared to SMOM2-expressing IFE cells. Furthermore, SMOM2 induced upregulation of Wnt/β-catenin signaling, as shown by increased nuclear β-catenin and lymphoid enhancer-binding factor-1 (LEF1) expression, which led to mice BCC and human BCC tumor initiation [66]. Besides BCC, follicular hamartomas, a rare benign tumor with the potential to develop into BCC, developed as a result of overexpressing the constitutive active SMOM2 mutant in transgenic mice revealing high levels of *GLI1* and *GLI2* transcripts in both in situ hybridization and northern blot analysis [62]. Taken together, these results confirm an SMO-dependent role of GLI regulation in BCC tumorigenesis.

Mutations in *PTCH1* and *SMO,* although to a lesser degree than BCC, have also been detected in other cancers such as medulloblastoma [54], mesothelioma [134], cervical cancer [61], breast cancer [57], odontogenic keratocystic tumors [55], acute lymphoblastic leukemia [56], and hepatocellular carcinoma (HCC) [68,135]. Similar to BCC, GLI proteins are commonly overexpressed in these cancers. Undoubtedly, mutation of Hh pathway upstream genes leads to the constitutive activation of GLI proteins, which is vital to the development and growth of these tumors. For instance, treating medulloblastoma cell lines and primary malignant pleural mesothelioma cultures with the SMO inhibitor cyclopamine significantly inhibited GLI1 expression and in vivo xenograft growth in nude mice [136,137], suggesting the importance of SMO-dependent GLI activation in the tumorigenesis of these cancers. 

In human HCC tumors, expression of SMO positively correlated with tumor size, while an inverse relationship was reported for PTCH1, suggesting overaction of Hh signaling as a result of SMO derepression. Notably, a novel *SMO* point mutation (A to T transversion at position 1723) was identified and associated with enhanced GLI1 expression in human HCC. Like human HCC tumor specimens, Hep3B had significantly higher levels of SMO than PTCH1, and treatment of Hep3B with KAAD-cyclopamine (antagonist of oncogenic mutant SMO) but not cyclopamine (antagonist of wild-type SMO) markedly repressed GLI1 activity, suggesting that genetic alteration of *SMO* can promote HCC carcinogenesis through GLI1 activation. Furthermore, KAAD-cyclopamine treatment suppressed the expression of the c-Myc proto-oncogene, a key oncogenic factor in hepatocarcinogenesis and a major regulator of cell proliferation, and inhibited Hep3B cell growth which suggests a role of SMO-mediated upregulation of c-Myc in enhancing HCC growth [68]. 

*PTCH1* LOH has been associated with enhanced medulloblastoma formation as a result of increased SMO and GLI activation [54], and the loss of GLI1 markedly suppressed spontaneous medulloblastoma formation in *Ptc1*^+/−^ mice. Interestingly, in a subset of *Ptc1*^+/−^ mice with the loss of GLI1 that still develop medulloblastoma, GLI2 expression was significantly upregulated and correlated with the levels of n-Myc, a regulator of D-type cyclins in neuronal cells, suggesting a compensatory GLI2/n-Myc expression in promoting medulloblastoma formation and proliferation [53]. These results suggest that dual inhibition of GLI1 and GLI2 is required to completely terminate all aspects of Hh signaling to inhibit medulloblastoma tumorigenesis.

Kadlub et al. reported that germline *PTCH1* mutations in one allele were found in all keratocystic odontogenic tumors of patients, except in one syndromic keratocystic odontogenic tumor that resulted from a somatic silent mutation. Based on the pathological findings, the authors proposed that mutation-harboring keratocystic odontogenic tumors contain a chorionic epithelial structure that serves as a germinal center that gives rise to new daughter cysts following the course of local inflammation, suprabasal proliferation, epithelial budding, chorionic epithelial island formation, and daughter cyst formation, leading to tumor recurrence [55].

Burns et al. reported Hh pathway mutations in 16% of childhood T-cell acute lymphoblastic leukemia (T-ALL), of which the majority of mutations were reported in the *PTCH1* gene [56]. Furthermore, Hh pathway mutations were associated with primary chemotherapy failure and increased incidence of relapse. Notably, transfection of the mutant *PTCH1* alleles identified in T-ALL specimens into the PTCH1-mutant T-ALL cell line Jurkat did not suppress growth in six out of seven of the tested mutant *PTCH1* alleles. Additionally, these mutants were impaired in their ability to suppress *GLI1* mRNA, while wild-type *PTCH1* induced apoptosis and suppressed *GLI1* expression. Furthermore, transduction of wild-type *PTCH1* into the *PTCH1*-mutant cells downregulated the expression of *MYCN*, which has been reported in approximately 20% of human T-ALL to drive oncogenic T-cell transformation [56]. 

To further corroborate these findings in vivo, coinjection of CRISPR/Cas9 cassettes targeting the exon 13 of *PTCH1* was shown to accelerate the onset of Notch1-induced T-ALL in a zebrafish model. Furthermore, treating zebrafish engrafted with T-ALL blasts harvested from the *PTCH1*-mutant zebrafish with the SMO inhibitor cyclopamine led to marked tumor regression, while no tumor growth inhibition was noted in zebrafish engrafted with T-ALL harvested from aavs1-control fish. Notably, treating primary leukemic cells from patient T-ALL D15 harboring the pathogenic *PTCH1* T1106M mutation with the FDA-approved SMO inhibitor vismodegib also effectively suppressed their viability and GLI1 expression [56]. These results confirm that *PTCH1* mutants must transduce Hh signals to GLI through SMO to induce tumorigenesis in T-ALL.

*PTCH1* mutations have also been reported in breast cancer patients, which were associated with poor prognosis and increased tumor recurrence rate [57]. Notably, high levels of Shh and GLI1 have been associated with the enhanced acquisition of CSC traits and chemoresistance in breast cancer, which can be attenuated with SMO inhibitors [58]. Thus, increased SMO-dependent GLI activation due to *PTCH1* mutation or Shh induction might promote tumor recurrence by supporting the formation of chemoresistant CSC niches in breast tumors. 

Deletion of *PTCH1* was also associated with the progression from early to advanced stages of cervical carcinoma and predicted poorer overall survival (OS) in patients. Additionally, lower expression of PTCH1 was positively correlated with increased nuclear GLI1 in both normal epithelium and tumor samples [60]. In a different study by Chaudary et al., high expression of *SMO*, as well as upregulation of more than 3 Hh genes, including *Shh*, *PTCH1*, and *GLI1*, was associated with increased incidence of local recurrence after chemoradiation [60]. A different study by Chen et al. also demonstrated that Shh and PTCH1 were significantly correlated with pathological tumor grade, as shown by enhanced expression of these proteins in poorly differentiated tissues [61]. Similarly, GLI1 was also expressed more in poorly differentiated tissues and was strongly correlated with tumor invasion and lymph node metastasis. Like GLI1, SMO was also strongly correlated with tumor invasion, although no association was found with the pathological tumor grade [61]. 

Of note, according to the TCGA pancancer atlas database, the frequency of *SMO*, *GLI1*, *GLI2*, and *GLI3* mutations and copy number variations (CNVs) is 2%, 3%, 3%, and 4%, respectively. Despite the lower frequency of mutations and CNVs reported in SMO compared to GLI proteins, SMO has the highest number of oncogenic driver somatic mutations reported at 4.8%, followed by GLI1 (1.9%), GLI2 (0.3%), and GLI3 (none). Nevertheless, oncogenic driver mutations in any upstream Hh components inevitably converge at GLI proteins, leading to the constitutive activation of GLI proteins. 

#### 3.1.2. Transcriptional and Epigenetic Regulation of Hh Pathway Genes Upstream of GLI

**Figure 5:**
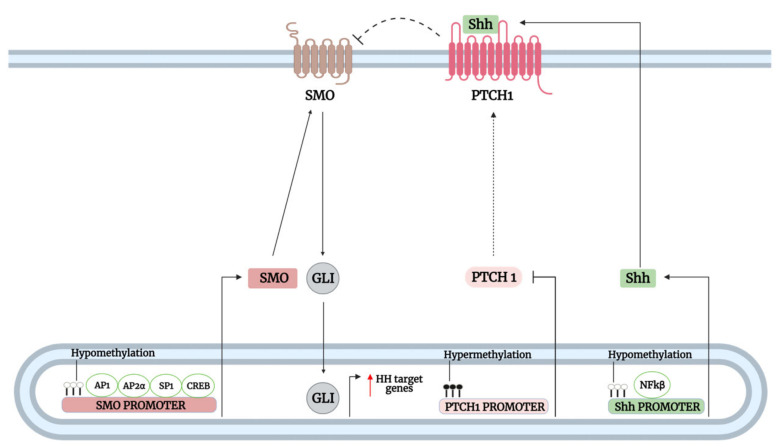
A simplified illustration of SMO-dependent GLI regulation in the context of transcriptional regulation. The binding of transcription factors AP1, AP2α, SP1, and CREB to promoter regions of *SMO* kickstarts the onset of its transcription. Similarly, the binding of NFκB to the NFκB binding site located within the *Shh* promoter induced the transcriptional upregulation of Shh. Furthermore, the increased transcriptional output of *SMO* and *Shh* can also occur as epigenetic events through hypomethylation of CpG islands that reside within promoters, which further promotes the binding of transcriptional machinery to promoters. Conversely, hypermethylation of the *PTCH1* promoter leads to decreased transcriptional expression and consequently decreased production of PTCH1 protein. Together, these molecular events work in concert to further enhanced Hh pathway activity and promote tumorigenesis. Dotted triangle-headed arrow: inactivated function; dotted bar-headed arrow: loss of inhibition; red upward triangle-headed arrow: upregulation.

Mutations in the Hh upstream elements are uncommon in many other tumors, indicating the existence of other dysregulated mechanisms affecting Hh-GLI signaling. Indeed, many of the mechanisms resulting in overactivation of SMO and subsequent activation of GLI result from the dysregulated expression of the upstream Shh/PTCH/SMO components of the Hh signaling pathway (Figure 5). Transcription factors bind to specific DNA consensus sequences within target gene promoters to initiate their transcription; however, when aberrantly activated, they lead to uncontrolled activation of target genes.

There is a lack of conclusive evidence to support *Shh* expression regulation by GLI transcription factors, but transcription factors external to the Hh pathway have been shown to regulate *Shh* expression at the promoter level. For instance, NFκB, a proinflammatory transcription factor, binds to the putative NFκB binding site in the *Shh* gene promoter to initiate its transcription in pancreatic carcinoma cell lines [69]. Increased Shh expression can promote the autocrine Hh-GLI pathway activation of cancer cells and stromal cells through repression of PTCH1 and increase SMO activation, thus leading to transcriptional activation of Hh target genes and carcinogenesis [72]. NFκB-mediated Shh upregulation was found to promote ASPC1 pancreatic cancer cell proliferation and protection against TRAIL-induced apoptosis/caspase 3 activation. Furthermore, ectopic induction of NFκB/IKK2 enhanced the expression of Shh in in vivo genetic mouse model and promoted pancreatic tumor growth in an in vivo chorioallantoic membrane tumor model, which can be reversed upon Shh silencing [69]. 

In further support of the findings above, Nakashima et al. also revealed a positive correlation between p65, the functional component of NFκB, and Shh expression in human PDAC tumor specimens [70]. Abundant amounts of p65 and Shh were also positively correlated in chronic pancreatitis specimens, suggesting a role of Shh signaling in promoting persistent inflammation that predisposes the development of cancer. By contrast, few to no detectable levels of p65 and Shh were found in normal pancreas specimens. In vitro study using cell lines revealed that NFκB upregulates Shh to induce the proliferation of ASPC1 and SUIT2 pancreatic cancer cells. Additionally, enhanced NFκB DNA-binding ability in these cell lines was associated with the constitutive expression of Shh, PTCH1, and GLI1 at both transcript and protein levels [70], suggesting an active Shh-GLI signaling axis.

Shh produced by pancreatic ductal epithelium can also upregulate *GLI1* mRNA in fibroblasts of the stromal compartment in a paracrine manner [71], and activation of canonical Hh-GLI signaling in stromal cells leads to paracrine feedbacks to the epithelial compartment, which, in turn, promotes pancreatic ductal adenocarcinoma (PDAC) progression and chemoresistance [72,73]. Notably, treating KrasG12D/+;LSL-Trp53R172H/+;Pdx-1-Cre (KPC) mice model with the SMO inhibitor IPI-926 enhanced stromal depletion and consequently improved gemcitabine delivery to PDAC tumor sites as a result of increased blood vessel perfusion [73]. Thus, high levels of NFκB in pancreatic epithelium may potentially enhance Shh expression to promote paracrine activation of Hh-GLI signaling in stromal cells, which in turn leads to desmoplastic stromal depletion, and decrease vascularization, which reduces gemcitabine delivery to tumor sites; however, this notion remains to be elucidated.

In 33 tumor cell lines, *SMO* gene expression was highest among all Hh members, and its expression was significantly and positively correlated with *GLI2* transcript levels. Key functional binding sites for CREB, AP1, AP2α, and SP1 transcription factors were identified in *SMO* promoter elements through luciferase reporter and electrophoretic mobility shift assay (EMSA), suggesting an important role of these transcription factors in *SMO* transcriptional activity and subsequent GLI2 activation in PC3 prostate cancer and MCF7 breast cancer cell lines [76]. Interestingly, Wnt3a treatment of human foreskin fibroblast cells induced the accumulation of cellular SMO and GLI proteins and the upregulation of SMO, PTCH, GLI1, GLI2, and GLI3 expression. Mechanistically, the β-catenin/T-cell factor 4 (TCF-4) complex directly binds to T/AC/GAAAG motifs residing in *SMO* and *GLI1* promoters, and the suppression of β-catenin downregulated SMO and GLI1 expression as well as foreskin fibroblast cell proliferation [77]. Whether this mechanism of SMO regulation is translatable to cancer cells remains to be explored.

Epigenetic mechanisms, such as DNA methylation, play a significant role in regulating the expression of genes, including members of the Hh pathway. For instance, hypomethylation of CpG islands on *Shh* promoter has been shown to facilitate the binding of NFκB to its site, in which its increased transcriptional activity led to enhanced self-renewal/colony formation and migration of breast cancer cell lines and could be inhibited by SMO inhibition with cyclopamine. Importantly, high levels of Shh expression in human breast cancer tissues were positively correlated with positive NFκB nuclear staining score, promoter hypomethylation, and worse OS, corroborating the oncogenic role of Shh in breast cancer [74]. Similarly, Cui et al. also reported hypomethylation of the *Shh* promoter region in more than half of the breast cancer carcinomas tissues, which was positively associated with nuclear GLI1 and NFκB expression [75]. Taken together, these results suggest hypomethylation of the *Shh* promoter facilitates the binding of NFκB to induce its transcription, and *Shh* upregulation leads to increased canonical Hh-GLI signaling by which enhanced SMO activation as a result of PTCH1 repression promotes breast carcinogenesis through GLI1 activation. 

Interestingly, Benvenuto et al. reported that SMO and GLI1 heavily regulated NFκB nuclear translocation [138], and together with the previous findings, suggested a positive feedback loop between Hh-GLI signaling and NFκB. Furthermore, treating BALB/*c* mice inoculated with mouse breast cancer cells with the SMO inhibitor GDC-0449 or GLI inhibitor GANT61 inhibited tumor growth in vivo by inhibiting Hh pathway activation, reducing cancer cell survival, and inducing apoptosis [138], confirming an oncogenic role of SMO-dependent GLI signaling in breast cancer tumorigenesis.

Hypomethylation of *SMO* promoter has also been reported to promote tumorigenesis through enhanced SMO-dependent GLI regulation. For instance, functional SMO is required for the expression of GLI3 in colorectal carcinoma cell lines, and promoter methylation of *SMO* led to decreased GLI3 expression. Conversely, treating colorectal cancer cells with demethylating agents 5-aza-20—deoxycytidine (5aza-dC) and TSA restored both SMO and GLI3 expression [78]. Of note, GLI3 overexpression in colorectal cancer cells has been implicated in enhanced colony formation, proliferation, and invasion by upregulating the expression of EMT factors (TWIST1, ZEB1, VIM, ZEB2, and CDH2) via positive regulation of ERK1/2 cascade, increasing the expression of adherence-related genes (*ITGA4*, *GDF15*, and *NXPH4*), and downregulating p53 levels. Furthermore, the GLI3-mediated tumorigenesis in several colorectal cancer cell lines, such as LOVO, HT29, and SW480 cells, were found to be dependent on active SMO and Shh signals [79,80,81]. Together with the role of functional SMO in regulating GLI3 activity, it suggests an SMO-dependent GLI signaling in colon cancer tumorigenesis. Hypomethylation of *SMO* promoter has also been detected in other cancers, including prostate, kidney, glioblastoma, and ovarian cancer cell lines, and a positive correlation was identified between *SMO* and *GLI2* transcript levels [76]. 

To further corroborate the importance of SMO in colorectal cancer tumorigenesis, Magistri et al. revealed that treating HCT 116, SW480, and SW620 colon cancer cell lines with SMO inhibitors or siRNA reduced cell proliferation via upregulation of p21 and downregulation of CCND1 and suppressed migration and three-dimensional invasion via downregulation of SNAI1 and induction of epithelial markers Cytokeratin-18 and E-cadherin. Similar findings were also obtained with the use of Shh inhibitor 5E1. In the same study, the authors found that GLI1 and GLI2 expression from a data microarray from a cohort of 382 colon cancer patients were strongly correlated with reduced OS and disease-free survival (DFS) [82]. In an earlier finding from the same group, inhibiting SMO in HCT 116 cells with SMO inhibitor resulted in deregulated cellular energetic metabolism, particularly the metabolism of nucleotide sugars, pyruvate, pyrimidine, and purine, and the citrate cycle and oxidative phosphorylation, which may potentially inhibit tumor growth [82]. In two independent studies, a positive correlation between Shh and GLI3 has also been reported in colorectal cancer tissues from patients [79,80]. Furthermore, implanting GLI3-expressing HT29 cells into severe combined immunodeficiency mice resulted in the development and enhanced growth of subcutaneous tumors [79]. Taken together, these findings strongly suggest an important role of the intact Shh-SMO-GLI axis in promoting colorectal cancer tumorigenesis.

Hypermethylation of the major negative regulator *PTCH1* gene also contributes to carcinogenesis by decreasing inhibitory feedback to the Hh signaling and increasing GLI activity through SMO derepression. Inhibition of DNA methyltransferase by 5aza-dC was found to reduce *PTCH1* DNA methylation, accompanied by decreased SMO and GLI1 expression and inhibition of GLI1 and GLI2 nuclear translocation in leiomyosarcoma cell lines. Consequently, decreased GLI activation resulting from increased PTCH1 inhibitory feedback led to decreased proliferation and migration while inducing apoptosis in the cell lines. Additionally, the blocking of SMO (LDE225) and GLI (GANT61) function by Hh inhibitors effectively reduced proliferation, migration, and invasion of leiomyosarcoma cell lines [83], and together with the previous results, suggested that PTCH1 downregulation can promote carcinogenesis of leiomyosarcoma cells through SMO derepression and subsequent GLI activation.

In a different study by Song et al., PTCH1 and hedgehog interacting protein (HHIP) transcript and protein levels were significantly downregulated in gastric cancer tissues, and subsequent analysis revealed significant promoter methylation of both genes [84]. Treating gastric cancer AGC cell line with 5aza-dC effectively reduced cell viability and induced apoptosis [84], suggesting a role of canonical Hh pathway activation in gastric tumorigenesis through hypermethylation and subsequent downregulation of the PTCH1 and HHIP negative regulators. Evidence for PTCH1 hypermethylation was also reported by Du et al. in a subset of gastric cancer patients [85]. In further support of the loss of PTCH1 in gastric tumorigenesis, Lee et al. reported that negative staining of PTCH1 in gastric cancer tissues of patients was positively correlated with reduced OS, while GLI2 was correlated with lymphovascular invasion [86].

In a study by Zuo et al., HHIP was hypermethylated in primary gastric cancer cells derived from two independent gastric cancer patients, and reversal of this methylation status or ectopically expressing HHIP inhibited their survival proliferation as well as migration and invasion [87]. Song et al. also reported marked lower HHIP levels in gastric cancer tissues compared to adjacent normal tissues, which was positively associated with gastric cancer metastasis [88]. Highlighting the importance of SMO in all these findings, Yang et al. revealed significantly elevated levels of SMO and GLI1 in gastric cancer tissues compared to normal paired tissues [89]. A study by Fukuya et al. also revealed elevated expression of Shh, PTCH1, SMO, GLI1, and GLI2 in the diffuse-type gastric cancer specimens compared to the intestinal-type gastric cancers [90]. Of note, diffuse-type gastric cancers have been reported to be more aggressive and metastatic than their intestinal counterpart, which suggests an association of Hh signaling with advanced stages of gastric cancer.

### 3.2. SMO-Independent GLI Activation

**Figure 6:**
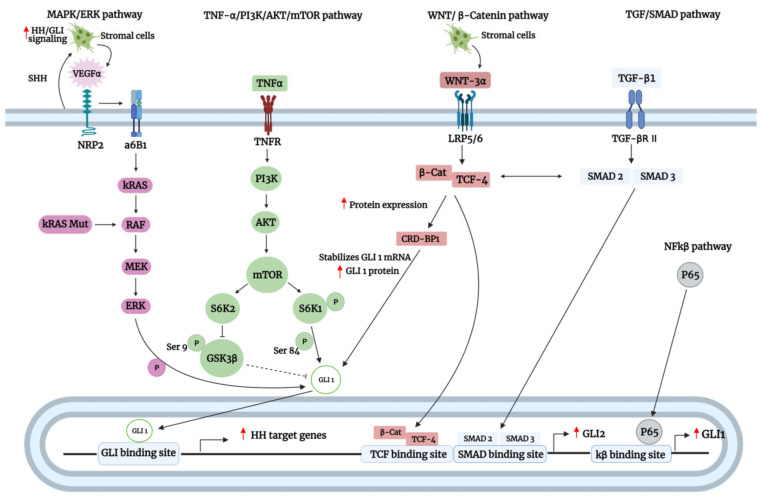
A schematic representation of the Smoothened (SMO)-independent regulation of glioma-associated oncogene homolog (GLI) transcription factors by oncogenic pathways. As shown above, GLI transcription factors can be regulated at the protein or transcriptional level depending on the oncogenic pathway involved. In the mitogen-activated protein kinase (MAPK)/ extracellular-signal-regulated kinase (ERK) pathway, sonic hedgehog (Shh) produced by tumor cells activates hedgehog (Hh)/GLI signaling in the stromal cells, leading to the upregulation of vascular endothelial growth factor A (VEGFa). Paracrine feedback of VEGFa to tumor cells is initiated upon binding of the VEGFa to neuropilin 2 (NRP2), which induces α6β1 integrin-mediated activation of kirsten rat sarcoma 2 viral oncogene homolog (KRAS)/ mitogen-activated protein kinase kinase (MEK)/ERK cascade. Active ERK1 then phosphorylates GLI1 protein, leading to its activation. Oncogenic *KRAS* mutations also lead to the constitutive activation of the MAPK/MEK/ERK pathway, consequently promoting GLI1 phosphorylation and activation. In the phosphoinositide 3-kinase (PI3K)/protein kinase B (AKT)/mechanistic target of rapamycin kinase (mTOR)pathway, tumor necrosis factor-alpha (TNFα) stimulation results in the activation of the mTOR complex, which in turn activates S6K2. Consequently, activated S6K2 phosphorylates glycogen synthase kinase 3 beta (GSK3β) at serine 9, leading to its inactivation. Inactivated GSK3β is not able to phosphorylate GLI1, relieving the inhibition of GSK3β on GLI1. Activation of the mTOR complex also activates S6K1 by phosphorylation, and activated S6K1, in turn, phosphorylate GLI1 at Ser9 to promote its activation. In the Wnt/β-catenin pathway, stromal cells produced Wnt3a that binds to the LRP5/6 receptor. The signal is then transduced to β-catenin, which forms a complex with T-cell factor 4 (TCF-4). The β-catenin-TCF-4 complex upregulates the protein expression of coding region determinant binding protein (CRD-BP), which stabilizes *GLI1* mRNA and consequently enhances GLI1 protein levels. In the transforming growth factor-β (TGF-B)/SMAD pathway, stimulation by TGF-β results in the activation of SMAD2/3. SMAD2/3 cooperates with the β-catenin-TCF-4 complex to upregulate the expression of *GLI2* by binding to the SMAD and TCF binding site within the *GLI2* promoter. In the nuclear factor kappa B (NFκB) pathway, the p65 subunit of the NFκB complex binds to the kB binding site within the *GLI1* promoter to initiate its transcription. Red upward triangle-headed arrow: upregulation.

**Figure 7:**
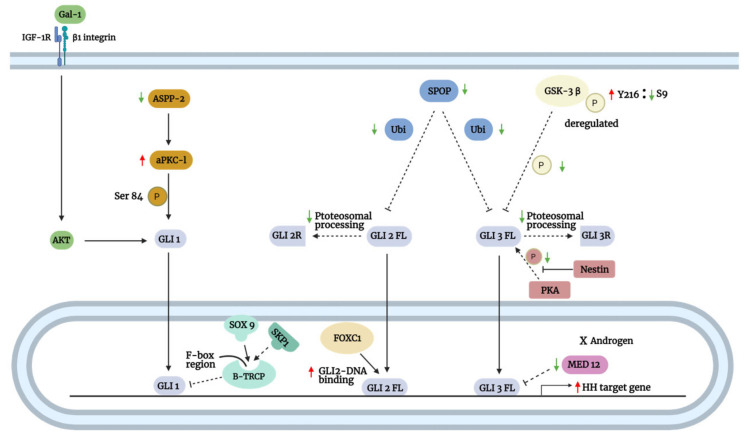
A schematic representation of the Smoothened (SMO)-independent regulation of glioma-associated oncogene homolog (GLI) transcription factors by their interacting proteins. Apoptosis-stimulating of p53 protein 2 (ASPP2) deficiency enhanced the binding of atypical Protein Kinase C ι (aPKC-ι) with GLI1, which allows aPKC-ι to phosphorylate GLI1 at Ser84. The phosphorylated GLI1 is, in turn, activated, promoting its translocation into the nucleus to transcribe target genes. Galectin-1 (Gal-1) binds to β1 integrin to promote GLI1 activation. Mechanistically, activated β1 integrin forms a complex with insulin-like growth factor 1 receptor (IGF-1R) to promote protein kinase B (AKT) activation, leading to an increase in GLI1 activity. In the nucleus, SOX9 binds to the F-box region of β-TrCP, interfering with its binding to SKP1. The binding of SOX9 to β-TrCP tethers it within the nucleus, thus protecting GLI1 from degradation. Speckle-type POZ protein (SPOP) downregulation results in decreased ubiquitination of full-length GLI2/3 proteins, favoring their activation and nuclear translocation over their proteasomal processing into repressors. In the nucleus, the N-terminal domain (aa 1-68) of transcription factor forkhead box C1 (FOXC1) binds to the internal region (aa 898-1168) of GLI2, enhancing its DNA-binding and transcriptional-activating ability. The imbalance between Tyr216 and Ser9 phosphorylation of glycogen synthase kinase 3 beta (GSK3β) leads to its dysregulated function, thereby impairing its ability to phosphorylate full-length GLI3 proteins. Unphosphorylated full-length GLI3 proteins are not subjected to proteasomal processing into their repressors, allowing their translocation into the nucleus to transcribe target genes. Under androgen-deprived conditions, the downregulation of MED12 relieves its constraint on the full-length GLI3 proteins, resulting in their hyperactivation. Green downward triangle-headed arrow: downregulation; red upward triangle-headed arrow: upregulation; dotted bar-headed arrow: loss of inhibition.

Numerous studies accounted for the involvement of various noncanonical mechanisms in the over-activation of GLI proteins, which explained the ineffectiveness of SMO and upstream inhibitors in the treatment of certain GLI-overexpressing cancers. These mechanisms involve the active crosstalk between the Hh pathway with multiple signaling pathways, including kirsten rat sarcoma 2 viral oncogene homolog (KRAS)/mitogen-activated protein kinase (MAPK)/extracellular-signal-regulated kinase (ERK), transforming growth factor-β (TGF-β)/SMAD, Wnt/β-catenin, phosphoinositide 3-kinase (PI3K)/protein kinase B (AKT)/mechanistic target of rapamycin kinase (mTOR), and nuclear factor kappa B (NFκB) signaling (Figure 6). Additionally, interacting proteins (e.g., kinases and transcription factors) can also regulate GLI noncanonically, independent of SMO (Figure 7).

#### 3.2.1. Active Crosstalk of GLI with Oncogenic Pathways

The interplay between GLI and oncogenic pathways is vital for the proper development and progression of cancers. For instance, it was shown that the KRAS/MAPK/ERK/GLI1 activation could be mediated by either oncogenic *KRAS* mutation or stimulation of neuropilin 2 (NRP2) by vascular endothelial growth factor (VEGF) in lung adenocarcinoma (LAC) of non-small cell lung cancer (NSCLC). In the latter, Shh paracrine crosstalk between the epithelial and stromal compartment of the LAC tumor triggers the canonical activation of the stroma Hh pathway. Consequently, this led to the increased production of VEGFa ligands by stromal cells, which interacted with the NRP2 receptor of the epithelial compartment to mediate noncanonical activation of GLI1 via the initiation of MAPK/ERK cascade. Mechanistically, in vitro kinase assay revealed that ERK1 phosphorylated GLI1 to regulate its transcriptional-activating ability. Furthermore, GLI1 inhibition by GANT61 or siRNA-mediated silencing inhibited LAC proliferation, attenuated CSC stemness feature and markers (OCT4 and ABCG2) and induced apoptosis in vitro and in vivo [91]. 

Importantly, this noncanonical route of GLI activation was frequently detected in patient-derived LAC CSCs. Notably, SMO was expressed at low levels in LAC cell lines and patient-derived LAC CSCs as a result of epigenetic silencing by hypermethylation, and together with the previous results, enforced a noncanonical role of MAPK/ERK in GLI1 regulation. Interestingly, the MAPK/ERK/GLI1 pathway could be further amplified by a positive feedback autocrine loop in which activation of the GLI1 resulted in the enhanced VEGFa expression and subsequent NRP2 function [91]. The lack of SMO expression in CSCs may partly explain the lack of benefit in lung cancer associated with the addition of SMO inhibitor to chemotherapy regimens, but there is yet to be a study to elucidate the importance of SMO/GLI in promoting chemoresistance in the context of CSC in lung cancer. Besides promoting stemness acquisition, high expression of VEGFa and NRP2 is associated with enhanced neovascularization of NSCLC tumors [92], which may suggest a role of GLI1 in mediating angiogenesis.

In further support of the findings above, Hh-GLI signaling in breast cancer can also be activated in a similar fashion. GLI activation upregulated the expression of VEGF and NRP2 in transgenic tumors of mouse models, which in turn induced α6β1 integrin-mediated activation of RAS/MEK signaling through focal adhesion kinase (FAK) activation to enhance GLI1 expression and promote tumor-initiating or CSC-like properties [93]. Interestingly, enhanced FAK signaling in tumor epithelium as a result of cancer-associated fibroblasts (CAF)-induced extracellular matrix (ECM) remodeling has been shown to confer CSC traits in in vitro and in vivo, which in turn enhanced chemoresistance to docetaxel. In the M6-Hh (Hh expressing murine triple-negative breast cancer) tumors, Shh expressed from tumor epithelium cells induced expression of GLI1 in CAFs of the surrounding stroma, resulting in their activation. In turn, activated CAFs remodel ECM to promote CSC traits in tumor epithelium via activation of their FAK signaling. SMO inhibition of the CAF cells reduced FAK signaling, FGF-5 expression, and CSC markers in M6-Hh tumors and patient-derived xenografts implanted in Rag^−^/^−^ and NOD-scid IL2rγnull mice, which in turn resensitized them to docetaxel. Moreover, in the phase-I EDALINE study, tumor specimens of high responders to docetaxel were cotreated with sonidegib and characterized by elevated Hh ligand expression, GLI1 expression, ECM remodeling, FAK signaling, phosphor-FGFR (receptor of FGF-5), and ALDH1 (CSC marker)-positive cells [58]. Taken together, these results suggest a cooperative role of SMO-dependent (stromal compartment) and SMO-independent (epithelial compartment) GLI activation in the activation of FAK signaling in breast tumor cells to confer CSC traits and consequently to enhance chemoresistance.

In another study, mutant KRAS drives PDAC tumorigenesis by regulating GLI1 expression independent of SMO. *KRAS* mutations are found in nearly all PDAC and are crucial drivers of PDAC growth [139]. The depletion of *KRAS* via siRNA-mediated knockdown led to the downregulation of GLI1 expression and the induction of mouse PDAC cell line apoptosis by caspase 3 activation. Similarly, *GLI1* knockdown also significantly induced human PDAC cell line apoptosis upon challenging it with cycloheximide, an inducer of programmed apoptotic cell death. However, inhibition of anchorage-independent cell growth was less profound in PDAC cells expressing wild-type KRAS compared to mutant KRAS, suggesting that GLI1 regulation is more accurately represented in the context of mutant KRAS. In support of this, the transfecting of oncogenic KRAS construct into PDAC cell lines expressing wild-type KRAS markedly enhanced their sensitivity to GLI1 knockdown. Moreover, the depletion of SMO had no impact on PDAC formation and GLI1 expression of PDAC transgenic mice model, and the stimulation with recombinant Shh did not affect *GLI* reporter activity, proving an SMO-independent mechanism of GLI regulation. Interestingly, downregulation of KRAS resulted in a significant reduction in the expression of GLI1 protein and vice versa, implying the existence of a self-sustaining loop between KRAS and GLI1 protein [94]. An in vitro study by Han et al. also revealed that an intact RAF-MEK1-ERK pathway was required for KRAS-mediated GLI1/2 activation in pancreatic cancer cells [140].

Rajurkar et al. have also shown a cooperative role between KRAS and GLI1 in promoting pancreatic tumorigenesis using in vivo mice models [95]. The authors demonstrated that GLI1 is required to mediate KRAS-induced survival and proliferation of primary pancreatic cells and KRAS-induced pancreatic intraepithelial neoplasia (PanIN) lesion and PDAC formation in vivo. Notably, KRAS-induced tumors with a loss of p53 were characterized by aggressive PDA cells that were more proliferative and metastatic with evidence of dissemination to lymph nodes, liver, lungs, peritoneal cavity, and adjacent intestine. Conversely, conditional Rosa26 knock-in allele of GLI3T, which was proven to downregulate GLI1 and GLI2 expression in NIH 3T3 cells, resulted in reduced PANIN lesion formation, reduced proliferative pancreatic cells (absence of Ki67 staining), and delayed PDAC tumor formation. Ectopic GLI1 expression in KRAS-expressing mice increased PanIN lesions’ formation, enhanced pancreatic cell proliferation as indicated by Ki67 staining, and promoted escape from growth arrest/senescence. Interestingly, ectopic GLI1 expression in KRAS-expressing mice enhanced IKBKE expression and nuclear RelA staining, and the knockdown of IKBKE expression in pancreatic cancer cell lines impaired their ability to grow in soft agar and induced apoptosis by caspase 3 cleavage [95]. Similar findings in a pancreatic cancer mouse model were also reported by Mills et al., who identified an additional IL-6/STAT3 axis regulated by GLI1 that was shown to be a crucial driver of KRAS-induced transformation [141].

TGF-β/SMAD signaling was also positively correlated with GLI1 and GLI2 protein expression across 26 types of tumors and did not share any prognostic value with other Hh-related genes, suggesting a role of noncanonical crosstalk between TGF-β/SMAD and GLI proteins [142]. TGF-β1 is a potent inducer of EMT in cancer, and treatment of hepatocellular carcinoma cell lines with TGF-β1 enhanced EMT acquisition via a GLI-SNAI1 dependent mechanism, which can be reversed by GLI1 knockdown. Furthermore, enhanced GLI1 expression was associated with enhanced colony formation, cell proliferation, viability, migration, and invasion in vitro, as well as enhanced vascular invasion in HCC biopsies [96]. Similarly, TGF-β1 can also induce Hh-GLI1 signaling to promote EMT phenotype and promote migration and metastatic characteristics in mesenchymal A549 LAC cells [143], which further supports the role of GLI in mediating TGF-β1-driven EMT in cancer. Such crosstalk between TGF-β and GLI protein has also been observed in other cancers, including melanoma [97] and PDAC [94]. 

Faião-Flores et al. reported a role of GLI1/2 in conferring resistance to BRAF inhibitor vemurafenib in melanoma [97]. Examination of melanoma patient specimens failing vemurafenib revealed that all were positive for GLI1 expression, while 40% were positive for GLI2 expression. Similarly, the induction of vemurafenib resistance in melanoma cell lines was associated with frequent elevated expression of GLI1, while expression of GLI2 was elevated but to a lesser extent. Further investigation revealed that TGF-β1/SMAD3 was directly involved in the noncanonical regulation of GLI1/2 activation in vemurafenib-resistant melanoma cell lines. Inhibition of GLI1/2 by GANT61 significantly blocked the ability of TGF-β1 to enhance colony formation of vemurafenib-resistant cells. Using a 3D reconstructed human melanoma skin model, both untreated naïve and resistant melanoma cells had elevated levels of MMP2/9 and were highly invasive, but treatment with GANT61, which downregulated GLI1/2 expression, reversed these effects. Both GANT61 and SIS3 (SMAD3 inhibitor) but not cyclopamine effectively suppressed GLI1/2 expression and decreased cell viability of vemurafenib-resistant cells by induction of apoptosis, as shown by the decreased Bcl2/Bax ratio. The downregulation of GLI1/2 was associated with increased microphthalmia transcription factor (MITF) expression and decreased epidermal growth factor receptor (EGFR) expression [97]. 

In an effort to determine the clinical relevance of the findings above in vivo, the relationship between GLI1/2, MITF, and EGFR was determined in the TCGA melanoma cohort. Indeed, GLI2 was found to play a major role in suppressing MITF expression in human melanoma specimens, and the expression of GLI1/2 was correlated with EGFR/AXL signatures. These findings were consistent with those of vemurafenib-resistant cells, which showed strong upregulation of GLI1/2 and EGFR and downregulation of MITF that can be reversed with GANT61. Importantly, inhibition of GLI1/2 could not only restore sensitivity to vemurafenib but also delay the onset of vemurafenib resistance and induce senescence in melanoma cells [97]. Taken together, TGF-β1/SMAD3 activates GLI1/2 via an SMO-independent mechanism to promote chemoresistance, enhance invasive potential, and promote the proliferation of melanoma. 

Wnt/β–catenin and TGF-β/SMAD signaling have been reported to cooperatively induce *GLI2* promoter activity by virtue of β-catenin/TCF-4 and SMAD binding sites found within the TGF-β responsive region (29–119 bp) of the *GLI2* promoter [144]. Interestingly, in highly metastatic MDA-MB-231 breast cancer cells, β–catenin/TCF-4 was shown to stimulate *GLI2* promoter activity by binding to TCF-binding element (TBE) within the promoter but only in the presence of active functioning TGF-β/SMAD signaling. Notably, when the SMAD binding site was mutated, overexpression of the β–catenin pathway failed to stimulate *GLI2* promoter activity, indicating Wnt and TGF-β signaling codependent regulation of *GLI2*. Similarly, TGF-β type II receptor (TGFBRII) deletion also reduced *GLI2* transcript levels [98]. 

Upon metastasis to bone, osteolytic tumors secrete parathyroid hormone-related protein (PTHrP) to induce bone resorption, triggering the release of TGF-β that promotes tumor growth. Indeed, human bone marrow HS5 and primary bone marrow stromal cells were found to be paracrine sources of Wnt3a ligands, and treatment of breast cancer MDA-MB-231 cells with conditioned media derived from these stromal cells enhanced GLI2 levels, which was also associated with an increase in PTHrP (osteolysis mediator) levels and bone destruction. Physiologically, primary bone marrow stromal cells markedly enhanced osteolytic lesion in vivo when coinoculated with MDA-MB-231 cells. Moreover deletion of β-catenin in MDA-MB-231 cells or Wnt3a in HS5 cells reduced the ability of HS5 cells to enhance bone destruction by MDA-MB-231 cells. Conversely, inhibition of LRP5/6 (receptor of Wnt3a ligand) with sclerotin reduced basal *GLI2* transcript levels in MDA-MB-231 cells, proving the existence of a paracrine Wnt signaling crosstalk between bone marrow stromal cells and tumor cells [98]. 

Not long after, a separate study revealed that GLI2 upregulated PTHrP expression and consequently bone invasion in oral squamous cell carcinoma (OSCC). Using an in vivo orthotopic model, human OSCC cell lines expressing higher levels of PTHrP were associated with enhanced bony invasion and bone destruction. Similarly, β–catenin overexpression and TGF-β treatment were also found to upregulate *GLI2* promoter activity and PTHrP levels, which in turn enhanced bone invasion and destruction. Conversely, the silencing of GLI2 in bony invasive human OSCC cells resulted in significantly lower expression of PTHrP and reduced bony invasion and bone destruction in vivo when injected into mice. In further support of these findings, higher levels of GLI2 in OSCC specimens of patients were associated with bony invasion in both qRT-PCR and immunohistochemistry analysis. Additionally, analysis of a cohort of 279 head-and-neck carcinoma datasets from cBioPortal also revealed significant coexpression of GLI2 and TGF-β1, which are of clinical relevance to the findings above [99]. Taken together, these studies suggest a cooperative role of Wnt/TGF-β1 signaling that converges on GLI2 to induce PTHrP expression and consequently bony invasion in cancer. 

Wnt signaling has also been reported to affect the expression of GLI proteins indirectly via an intermediate. For instance, the β–catenin/TCF-4 complex upregulates the expression of RNA-binding protein, the coding region determinant binding protein (CRD-BP). CRD-BP can bind to the coding region of *GLI1* mRNA, thereby stabilizing its steady-state transcript and protein levels. Furthermore, GLI1 contributed to Wnt/βcatenin–dependent proliferation of human colorectal cancer cells, and GLI1 transfection into cancer cells could rescue colony formation when Wnt/β–catenin signaling was inhibited. Notably, a PCR analysis of primary human colorectal tumor samples that were previously shown to activate β-catenin and express high levels of CRD-BP was characterized by high levels of *GLI1*, which was consistent with those in a panel of established colorectal cancer cell lines [100]. 

Additionally, c-Myc, a key target of β–catenin, can both positively and negatively regulate the expression of GLI1A and GLI3R, respectively, which subsequently enhance Hh signaling in colon carcinoma cell lines. Interestingly, endogenous *c-Myc* can also be upregulated by endogenous GLI1 expression, suggesting a positive transcriptional regulatory loop between c-myc and GLI1. Furthermore, GLI1 overexpression rescued the inhibition of *c-Myc* and colony formation by a dominant-negative TCF-4 [101]. Astonishingly, other studies have also reported an antagonistic effect between the Hh and Wnt/β-catenin signaling. For instance, the overexpression of β-catenin reduced butyrate-induced GLI1 overexpression in gastric cancer cells [145], making their crosstalk in cancers more perplexing than it seems. 

Numerous studies have implicated a noncanonical role of PI3K/AKT/mTOR signaling in regulating GLI proteins in multiple cancers. For instance, GLI expression in gastric cancer was shown to be heavily modulated by phospho-AKT (p-AKT) activity. Notably, the expression of p-AKT was positively correlated with GLI1 expression in human gastric cancer tissues, with a stronger correlation in advanced gastric cancer stages; higher levels of p-AKT and GLI1 were also detected in advanced stages of gastric cancer. Additionally, high expression of both p-AKT and GLI1 was significantly associated with the following clinicopathological factors: tumor size, lymph node metastasis, invasion depth, venous invasion, degree of differentiation, and TNM staging. Evidently, p-AKT knockdown decreased GLI1 protein expression without affecting SMO levels, and this was associated with depressed growth and migration and enhanced cisplatin sensitivity of gastric cancer cell lines. Consistent findings were also revealed upon further investigation in vivo, where the concurrent inhibition of GLI and PI3K/AKT/mTOR signaling in mouse subcutaneous xenograft model enhances tumors’ sensitivity to cisplatin, as indicated by reduced tumor burden [102].

Interestingly, Chakrabarti et al. demonstrated that GLI2 promoted the expression of programmed death ligand-1 (PD-L1) to inhibit CD8+ cytotoxic T lymphocyte (CTL) cells’ effector function in gastric cancer [103]. Treatment of *iLgr5; GLI2A* mice with GANT61 resulted in the loss of tumor formation, reduced proliferation of the gastric epithelium as indicated by decreased PCNA staining, and reduced CD8+ CTL cells infiltration within tumors. Coculturing of *iLgr5; GLI2A* mice-derived organoids with CTLs and dendritic cells revealed that tumor antigens secreted from the cancer organoids are presented by dendritic cells to induce PD-1 expression on CTL cells. However, significant CTL-induced organoid apoptosis could only occur when cocultures were pretreated with PD-L1 inhibitors, indicating that PD-L1 expressed on cancer organoids inactivated CTL cells’ effector function by interacting with PD-1 expressed on CTL cells. Additionally, patient-derived gastric cancer organoids expressing high levels of GLI2 were found to be highly resistant to chemotherapeutic drugs epirubicin, oxaliplatin, and 5-fluorouracil compared to those without GLI2 expression in both in vitro and in vivo, while treatment with GANT61 resensitized the organoids to chemotherapy [103]. Further study by the same group revealed that the PI3K/AKT/mTOR pathway activated in patient-derived organoids noncanonically upregulated GLI1 and GLI2 to induce PD-L1 expression, which could be effectively suppressed with rapamycin [104]. Taken together, GLI1/2 mediates mTOR-induced PD-L1 expression to promote immune evasion of cancer cells, as well as promotes chemoresistance.

Kasiri et al. reported a trend where high *GLI1* transcript expression in NSCLC patients was associated with worse OS [105]. Additionally, the loss of GLI1 by shRNA-mediated knockdown significantly suppressed cell proliferation of subcutaneous SCC xenograft tumors in vivo. In vitro study demonstrated that inhibition of PI3K/mTOR signaling effectively diminished GLI1 expression and inhibited clonogenicity and proliferation of lung squamous cell carcinoma cell lines. Likewise, regulation of GLI1 was also independent of canonical Hh signaling, as neither SMO inhibition by GDC-0449 nor induction by SAG had a significant impact on both GLI1 transcript and protein levels; SMO inhibition also did not affect colony formation and cell proliferation. The concurrent inhibition of GLI and PI3K/AKT/mTOR signaling demonstrated a synergistic effect in inhibiting in vivo cancer cell growth, evident by reduced tumor burden of xenografts compared to treatment with any of the agents alone [105]. GLI1 expression has also been reported to be regulated by PI3K/AKT/mTOR signaling in several other cancers to promote tumorigenesis, including esophageal adenocarcinoma [106], melanoma [113], osteosarcoma [107], pancreatic cancer, ovarian cancer [108], and renal cancer [109].

S6K1/2, members of the ribosomal S6 kinase family, are downstream targets of PI3K/AKT/mTOR and are involved in protein synthesis and cell proliferation. Notably, their activation has been linked to increased GLI1 expression and activity in multiple cancers. Tumor necrosis factor-alpha (TNFα) induced SK61 phosphorylation, which was associated with enhanced GLI1 expression and GLI1 target genes, including cell cycle regulators *CCND1* and *n-Myc*, in prostate cancer PC3 cells. Consistent with the upregulation of these genes, GLI1 depletion by either GANT61- or siRNA-mediated knockdown effectively suppressed PC3 cell viability, liquid colony formation, and cell proliferation. Conversely, genetic and pharmacological inhibition of PI3K/mTOR inhibited TNFα-induced SK61 phosphorylation and consequently GLI1 expression, which led to decreased PC3 cell viability [110]. Interestingly, a study by Wang et al. reported that in esophageal adenocarcinoma cell lines, TNFα stimulation and ectopic SK61 expression regulate GLI1 activity by phosphorylation of its Ser84 residue, thereby dissociating GLI1 from SUFU and allowing GLI1 translocation into the nucleus [111]. Conversely, inhibition of SK61 activation by PI3K/mTOR inhibitor rapamycin and RAD-001 enhanced HH inhibitor GDC-0449 cytotoxic effect in both in vitro and in vivo models. Additionally, GLI1 was required for TNF3/mTOR/S6K1-mediated cell proliferation, as GLI1 knockdown abrogated TNFα- and S6K1-induced cell viability, proliferation, and invasion. Of note, SMO inhibition with cyclopamine or siRNA-mediated knockdown does not interfere with TNFα-induced GLI activity, suggesting an SMO-independent regulation of GLI1 activity by TNFα [111]. 

P70S6K2, another known downstream effector of the PI3K pathway, was also reported by Mizuarai and colleagues to regulate GLI1 expression through modulation of GSK3β, a negative regulator of GLI activation. Evidently, p70S6K2 inhibited GSK3β function by phosphorylating its Ser9 residue, which in turn stabilized GLI1 protein levels and enhanced cell viability/proliferation of non-small cell lung cancer cell lines, and this effect can be reversed upon p70S6K2 silencing. The G1/S cell-cycle-regulator gene CCND1 and apoptosis-inducer gene γ-catenin were markedly downregulated and upregulated, respectively, by siRNA-mediated knockdown of p70S6K2 in a dose-dependent manner, mirroring that of GLI1 inhibition [112].

Lastly, other routes of noncanonical GLI regulation also include NFκB signaling. In claudin-low breast cancer and EMT cell lines, the p65 subunit of the NFκB act as a transcriptional regulator of *GLI1* expression by binding to the κB binding site located within the *GLI1* promoter. Indeed, *NFκB* knockdown resulted in a significant decrease in GLI1 expression, and GLI1 knockdown resulted in decreased claudin-low breast cancer and EMT cell lines’ tumorigenicity. Additionally, GLI1 knockdown in claudin-low breast cancer cell lines significantly attenuated cell proliferation, migration, anchorage-independent growth, self-renewal, and reduced tumor xenograft growth in vivo. By contrast, treatment with several SMO inhibitors does not affect *GLI1* transcript levels or proliferation of EMT cell lines, indicating SMO-independent activation of GLI1 by the NFκB pathway [114].

Of note, osteopontin, a bone matrix protein, has been shown to noncanonically activate GLI1 in breast cancer to promote the acquisition of mesenchymal phenotype via upregulation of mesenchymal (N-cadherin, vimentin, TWIST, and SLUG) and downregulation of epithelial (E-cadherin and keratin-18) markers, as well as promote drug resistance to doxorubicin, paclitaxel, and cisplatin via upregulation of ABC transporters (ABCB1 and ABCG2). Interestingly, inhibition of both SMO and osteopontin resulted in greater suppression of the expression of ABC transporters [146], which suggests a cooperative role of both SMO-dependent and SMO-independent axis in promoting breast cancer chemoresistance. As mentioned previously, NFκB has also been shown to transcriptionally enhance *Shh* expression to promote breast cancer tumorigenesis in two independent studies [74,75], and Shh induction promoted chemoresistance of breast cancer by forming CSC niches [58]. Thus, GLI1 activation by SMO-dependent or -independent mechanisms in breast cancer cells could promote EMT-like changes and stemness acquisition, resulting in enhanced metastatic potential and chemoresistance. 

#### 3.2.2. Active Crosstalk of GLI with Oncogenic and Tumor Suppressor Proteins

Apoptosis-stimulating of p53 protein 2 (ASPP2), a haploinsufficient tumor suppressor of the ASPP family, is frequently downregulated in multiple types of cancers, leading to increased tumor metastasis. In gallbladder cancer, ASPP2 deficiency was found to mediate tumor invasion and metastasis through the aPKC-ι/GLI1 pathway. Clinically, reduced expression of ASPP2 was positively correlated with advanced TNM stages, poor tumor differentiation, and lymph node metastasis [126]. aPKC-ι has been shown to regulate GLI activity in BCC and function downstream of SMO [147]. Similarly, ASPP2 depletion in gallbladder cancer cell lines was shown to enhance the expression and binding of aPKC-ι with GLI1. Consequently, aPKC-ι phosphorylated GLI1 Ser84, which, in turn, promoted its nuclear translocation to activate cytokine genes (*CCL2*, *CCL5*, and *TNFα*) involved in the recruitment of tumor-associated macrophages (TAMs). Consequently, increased TAMs recruitment promoted EMT-like changes in gallbladder cancer tissues and enhanced their tumor lung metastases in vivo. Furthermore, coculture of gallbladder cancer cells with macrophages or a macrophage-derived conditioned medium enhanced cell migration with a concomitant increase in mesenchymal N-cadherin and vimentin and a decrease in epithelial marker E-cadherin. Interestingly, GLI1 can also regulate *PRKCI* (gene encoding aPKC-ι) by directly binding to its promoter region, implying the existence of a positive feedback loop. Of note, SMO inhibition by both cyclopamine and siRNA-mediated knockdown had no significant effect on GLI1 expression in gallbladder cancer cells, suggesting SMO-independent, aPKC-ι-mediated GLI1 activation [126]. 

Transcription factor SOX-9, a novel cancer stem cell marker, expression was shown to be regulated by GLI1 to promote CSC features in PDAC PANC-1 cells. The CSC spheroids were enriched for GLI1 regulatory genes (*GLI1*, *GLI2*, *SOX9*, and *SNAI1*) and pancreatic CSC markers (*CD24*, *CD44*, *ESA*, *CD133* and *CXCR4, OCT4,* and *KLF4*). The suppression of either SOX-9 or GLI1 impaired CSC markers’ expression with very similar profiles. Additionally, SOX9 suppression significantly impaired spheroid formation and side population cells. SOX9 suppression also significantly attenuated PDAC cell proliferation, anchorage independence, and survival. Conversely, restoring GLI1 expression by siRNA-mediated knockdown of β-TrCP, a negative regulator of GLI1, rescued cell death induced by SOX9 deficiency. Conversely, cosuppression of GLI1 and SOX9 further enhanced cell death [115]. 

Mechanistically, SOX9 inhibited the function of β-TrCP, a negative regulator of GLI, by binding to specific protein motifs (F-box region) present in the β-TrCP subunit, thereby disrupting its interaction with GLI protein. Additionally, SOX9 interfered with the β-TrCP function by blocking its interaction with SKP1, an essential subunit of the SCFβ-TrCP complex, and tethering it within the nucleus of PDAC cells to protect nuclear GLI1 from degradation. Notably, a positive feedback loop between SOX9 and GLI1 has been reported. To provide greater clinical relevance to human physiology, the authors assessed the expression of SOX9 and β-TrCP expression in primary human PDAC specimens by utilizing Oncomine microarray data, and as seen in their studies, *SOX9* mRNA upregulation was accompanied by downregulation of *BTRC* (encodes for β-TrCP), suggesting a potential for SOX9-mediated GLI1 upregulation via downregulation of β-TrCP [115].

Transcription factor forkhead box C1 (FOXC1), a known inducer of oncogenesis in breast cancer, has been reported to be overexpressed in basal-like breast cancer (BLBC) to promote CSC traits. Notably, increased levels of FOXC1 were associated with the upregulation of GLI2 protein accompanied by an increased BLBC stem-like phenotype. FOXC1-mediated upregulation of ALDH1 activity and mammosphere formation capacity was significantly attenuated by GLI2 knockdown in MDA-MB-231 cells. Interestingly, the ectopic expression of mouse GLI2, whose expression was not affected by GLI2 shRNA, could rescue the GLI2-knockdown-induced decrease in ALDH activity and mammosphere growth in GLI2 knockdown FOXC1-overexpressing MDA-MB-231 cells. Mechanistically, it was shown that the internal region (aa 898–1168) of GLI2 was a direct-binding site for the FOXC1 N-terminal domain (aa 1–68), which contributed to GLI2-enhanced transcriptional-activating capacity. In support of this, FOXC1 enhanced the DNA-binding ability of GLI2, evident by enhanced binding of GLI2 at the promoter of *FAM38B* gene, which is also markedly upregulated in breast cancer cells as a result of FOXC1 overexpression. Moreover, GLI1 (GANT61) but not SMO (GDC-0449 and LDE225) inhibition significantly reduced FOXC1-induced GLI-BS-luciferase activity in both SMO-positive and SMO-negative breast cancer cell lines, suggesting an SMO-independent activation of GLI [116].

To confirm the findings above in vivo, the authors injected FOXC1-overexpressing MDA-MB-231 cells orthotopically into the fourth mammary glands of BALB/c nude mice, which led to tumor development. Conversely, injected FOXC1-knockdown cells halted tumorigenesis. Additionally, FOXC1-overexpressing BLBC cells were less sensitive to the inhibitory effect of SMO inhibitor GDC-0449 compared to vector-overexpressing BLBC cells both in vitro and in vivo. Notably, establishing GD-0449-resistant BLBC sublines by long-term culturing of parental cells in the presence of escalating dose of GDC-0449 was accompanied by enhanced FOXC1 expression, suggesting a compensatory FOXC1-mediated upregulation of GLI that was independent of SMO activity. In clinical samples of BLBC patients, most were evident for the expression of FOXC1 and GLI2, and a strong correlation was noted between the expression of the two proteins. Similarly, an analysis of breast cancer samples from the TCGA database and Curtis dataset also revealed a strong correlation between FOXC1 and GLI2. Importantly, upregulation of Hh pathway-associated genes in patients was significantly correlated with FOXC1 expression and decreased DFS compared to patients not enriched for Hh pathway-associated genes [116].

Speckle-type POZ protein (SPOP), an E3 ubiquitin ligase substrate-binding adaptor protein, was markedly downregulated in human ovarian cancer tissues, and its lower expression levels were associated with advanced cancer stages and malignancy. SPOP transfection was associated with decreased proliferation, colony formation, and enhanced apoptosis of OVCAR-3 ovarian cancer cells, while SPOP knockdown produced the opposite effects. Additionally, SPOP transfection downregulated proliferating cell nuclear antigen (PCNA) and antiapoptotic protein BCL2 and upregulated proapoptotic proteins cleaved-caspase3 and Bax expression, while SPOP knockdown produced the opposite effects. Interestingly, low SPOP levels contributed to enhanced Hh signaling by modulating GLI1/2 expression, resulting in decreased apoptosis in ovarian cancer cell lines [123]. 

In a separate study by Zeng et al., SPOP was also significantly depleted in gastric cancer tissues compared to adjacent gastric mucosa tissues [124]. An analysis of clinicopathological features revealed a negative correlation between SPOP and lymph node metastasis, poor tissue differentiation, and advanced TNM stages. SPOP transfection of gastric cancer cell lines inhibited cell proliferation, migration, and colony formation. Mechanistic studies revealed that SPOP inhibited Hh pathway activation by promoting the proteasomal-dependent degradation of GLI2 in the cytoplasm. Likewise, SPOP repression enhanced GLI1/2 levels, which consequently promoted gastric cancer cell proliferation and migration as well as attenuated apoptosis. The transfection of SPOP was associated with upregulation of GLI1 regulatory targets, including caspase 3, cleaved caspase 3, and PARP, while GLI1 knockdown downregulated caspase-3, cleaved caspase-3, tumor suppressor PTEN, and cyclin-dependent kinase inhibitors p16, p21, and p27 and upregulated pro-proliferative proteins, including cyclin B1 (CCNB1), PCNA, p-ERK, and Daxx [124]. In support of the findings from Zend et al., Wang and colleagues have also demonstrated that SPOP interacted with the C-terminal region of GLI2 to promote its proteasomal-dependent degradation in C3H10T1/2 cells, and SPOP knockdown restored the levels of GLI2 [125]. 

Nestin, an important biomarker of stem cells, has been used to identify cells with cancer stem-like phenotype in a CSC population. Nestin was shown to play a crucial role in medulloblastoma-like tumor formation in transgenic mice with deleted PTCH1 in cerebellar granule neuron precursor cells, and its knockdown markedly impaired proliferation and induced differentiation in vitro, but unlike the function of Nestin, no stem cell properties were observed. Furthermore, Nestin knockdown in medulloblastoma cells significantly inhibited proliferation in vitro and in vivo, independent of apoptosis. Mechanistically, Nestin promoted tumorigenesis by modulating GLI3 activity and consequently Hh pathway activity. Nestin interacted with the C-terminal region of GLI3 to prevent its phosphorylation (presumably by PKA) and proteolytic processing into repressor form, thereby impairing its role as a negative regulator of the Hh pathway [117]. 

GSK3β is an established inhibitor of full-length activator GLI and is known to promote GLI2/3 processing into their repressors via phosphorylation of serine residues residing in GLI proteins. Interestingly, Trnski et al. reported high levels of GSK3β and GLI3 in colon cancer tissue specimens, while SMO and PTCH1 were only detected in less than half the samples [127]. GSK3β expression was significantly associated with higher DUKES’ stage and lymph node involvement, with a similar trend observed for GLI3. In vitro study revealed that GSK3β was positively correlated with colon cancer cells’ survival, and their higher expression levels were unexpectedly associated with enhanced Hh-GLI signaling. Reduced GLI1 expression due to inhibition of GSK3β activity with lithium chloride significantly inhibited cell proliferation and induced apoptosis, which was associated with enhanced caspase 3 and PARP cleavage. Unlike GLI, SMO inhibition with cyclopamine had no effect on cell proliferation and had a minimal effect on GLI1 expression [127]. 

Further investigations revealed that the inhibitory Ser9 phosphorylation of GSK3β was largely absent in colon cancer cell lines, whereas the activating Tyr216 phosphorylation remains, suggesting deregulated GSK3β function. Intriguingly, treating colon cancer cell lines with lithium chloride, a GSK3β inhibitor, increased Ser9 phosphorylation of GSK3β, which in turn restored the balance between the activating Tyr216 and inactivating Ser9 phosphorylation on GSK3β and restored its proper function. Consequently, the restoration of GSK3β proper function accelerated the formation of the GLI3-SUFU-GSK3β complex, which led to more efficient processing of GLI3 into its repressor form and consequently Hh-GLI signaling downregulation [127]. This study also suggests that imbalance activation and inactivation phosphorylation of GSK3β lead to its impaired function and, as a consequence, enhance Hh-GLI signaling and survival of colon cancer cells. 

In prostate cancer cells, it was reported that the *MED12* loss of function mutation promoted the androgen-independent cell growth of prostate cancer cells. Castration-resistant prostate cancer (CRPC) is known to develop as a result of androgen deprivation treatment. In this study, knockdown of MED12 was found to relieve its constraint on the GLI3-protein in the absence of androgen, leading to hyperactivation of GLI3 and consequently androgen-independent growth of LNCaP cells [128]. Notably, Zhou et al. revealed that overexpression of the GLI3-binding domain on MED12 inhibited the transactivation function of GLI3 [36], recapitulating the effect of the loss of MED12 on GLI3 hyperactivation in CRPC. 

High expression of Galectin-1 (Gal-1) was associated with increased invasion and metastasis of gastric cancer cells and a poorer prognosis and survival, as well as increased lymph node metastasis and tumor invasion depth in gastric cancer patients. In human GC tissue samples, a positive correlation between Gal-1 and GLI1 was noted; it was shown that Gal-1 upregulated the expression of GLI1 through an SMO-independent manner [118]. Mechanistically, Gal-1 secreted by gastric cancer tissue-derived CAFs binds to β1 integrin, an important regulator of cancer cell invasion, to induce GLI1 expression and gastric cancer cell invasion, while siRNA-mediated knockdown of β1 integrin abrogated this effect [119]. In further support of this, Goel et al. reported that β1 integrin regulated GLI1 activity through the modulation of the insulin-like growth factor 1 receptor (IGF-1R)/AKT pathway [148]. β1 integrin forms a complex with IGF-IR to promote AKT activation, leading to an increase in GLI1 expression [148]. The siRNA-mediated knockdown of GLI1 could also abolish Gal-1-induced gastric cancer invasion and expression of mesenchymal markers, including N-cadherin, vimentin, and SNAI1, and invasion-related protein MMP9, as well as restore the expression of the epithelial marker, E-cadherin [119]. 

Similarly, Gal-1 was also reported to modulate the expression of GLI1 in PDAC to promote proliferation, angiogenesis, and desmoplasic reaction. In *ela-myc* mice model, Gal-1 was mainly restricted to the stromal compartment, and Gal-1 knockout enhanced mice survival and impaired pancreatic tumor proliferation. Furthermore, the loss of Gal-1 impaired acinar-to-ductal metaplasia, an important step that precedes the transition to PDAC. Additionally, Gal-1 could enhance the immune cell evasion of pancreatic tumors by hampering T-cell and neutrophil tumor infiltration but whether GLI1 mediated this effect remains to be elucidated. Importantly, Gal-1 could enhance GLI1 expression in both fibroblast and tumor epithelium cells. As Gal-1 is restricted to the stromal compartment as assessed in pancreatic tumor specimens, Gal-1 could potentially induce GLI1 expression in tumor epithelium cells via paracrine induction, recapitulating the importance of stromal-derived Gal-1 in inducing GLI1 expression in the surrounding tumor [122]. 

Interestingly, Gal-1 was found to induce vasculogenic mimicry (VM), an endothelial-independent vascular system that allows blood transportation, through GLI1-mediated upregulation of VM-related molecules, including MMP2, MMP14, and laminin5γ2, in gastric cancer cell lines and tumor xenografts [120]. Immunohistochemical analysis of gastric cancer tissues revealed a significant correlation of Gal1 and GLI1 expression, and GLI1 expression was significantly correlated with VM. In an earlier study by the same group, immunohistochemical analysis of human gastric tissues revealed that Gal-1 expression was significantly correlated with vimentin, a mesenchymal marker shown to be positively correlated with VM. Further investigation revealed that Gal-1 promoted VM formation via activation of the EMT pathway to promote gastric cancer progression [121]. As Gal-1 was shown to modulate GLI1 expression to promote EMT [118,119], Gal-1 may have GLI1 as a mediator of the EMT pathway to induce VM in gastric cancer.

## 4. Hh Pathway as Therapeutic Targets in Cancer Clinical Studies

Since the discovery of the Hh pathway aberrant activation in cancers, tremendous efforts have been made to develop new Hh pathway targeting agents for the treatment of malignancies. From a wide range of Hh inhibitors that were developed and tested preclinically, SMO and GLI inhibitors have shown the most promise due to their improved efficacy in both in vitro and in vivo cancer models. In light of these findings, several Hh inhibitors, including the cyclopamine derivatives vismodegib, sonidegib, glasdegib, taladegib, saridegib, and TAK441, the antifungal itraconazole (also inhibits SMO), and the GLI inhibitor arsenic trioxide, are currently being developed in clinical studies. In particular, due to the durable objective tumor response rate (ORR) backed by Hh signaling’s well-defined pathological role in BCC and acceptable toxicity in the tested populations, vismodegib and sonidegib have been recently approved by the FDA for the treatment of adults with BCC who are not candidates for surgery or radiation [149,150]. Clinical investigations are currently ongoing for these drugs in other solid tumors and hematologic malignancies and their utility in combination therapy. Like vismodegib and sonidegib, glasdegib has also recently been approved by the FDA for the treatment of adults with acute myeloid leukemia (AML) who are not candidates for intensive induction chemotherapy [151]. Due to its promising development in the treatment of AML, glasdegib is currently being tested in phase III, randomized (1:1) double-blind global trials (BRIGHT AML 1019; NCT03416179) for its utility in combination with intensive (glasdegib plus cytarabine and daunorubicin) or nonintensive (glasdegib plus azacitidine) chemotherapy [152].

By contrast, despite the superior pharmacological properties of GLI inhibitors, arsenic trioxide remains the sole GLI inhibitor that has been investigated clinically in Hh pathway-active cancers such as BCC (NCT01791894, completed) [153]. Nonetheless, the frequent development of SMO inhibitor resistance (discussed in Section 3.1.1) in advanced BCC still warrants the development of new GLI inhibitors and investigation of their potential use as second-line therapy in clinical settings. Additionally, GLI inhibitors are more suitable candidates for treating noncanonical Hh pathway-associated cancers that are not dependent on SMO input for GLI activation (discussed in Section 3.1.1 and Section 3.1.2). Xie et al. have described clinical trials of Hh inhibitors in cancer treatment published from 2013–2017 [154]; thus, we summarized the latest clinical findings concerning the use of Hh inhibitors in cancer treatment published from 2017–2021 (Table 2).

To date, BCC remains the most clinically relevant cancer associated with the Hh pathway. BCC is rarely fatal but, in some cases, may develop into an advanced or invasive type of BCC when left untreated. There are two categories of advanced BCC: locally advanced BCC (laBCC) and metastatic BCC (mBCC). In clinical trials, laBCC tumors are characterized by at least one histologically confirmed lesion ≥10 mm that is either inoperable or has recurred following surgery or radiotherapy, while mBCC tumors are characterized by histologically confirmed BCC that has metastasized to distant organs, such as the lung, liver, lymph nodes, or bones (NCT01367665) [163,165,166,167].

As most BCC exhibit mutation in the *PTCH1* gene that results in SMO hyperactivation [130], they are largely amenable to treatment with SMO inhibitors. Vismodegib has been FDA approved for the treatment of laBCC and mBCC in 2012. The FDA approval was based on favorable results from a phase II clinical trial study in which vismodegib exposure led to a 30% ORR in mBCC and 43% ORR, including 21% complete response (CR) in laBCC [194]; the long-term follow-up reported a better ORR of 48.5% in mBCC and 60.3% in laBCC [162]. Not long after, sonidegib was FDA approved in 2015 for the treatment of laBCC after displaying ORR comparable to vismodegib in a phase II clinical trial [195]. Moreover, the utility of SMO inhibitors, including sonidegib and vismodegib, has proven to be clinically beneficial for the treatment of nonadvanced BCCs in NBCCS patients [176] and for the long-term intermittent treatment of multiple nonadvanced BCCs, including those with NBCCS [160], which warrants further study. In a phase II clinical trial study, topical patidegib consisting of the IPI-926/saridegib SMO inhibitor has recently shown promise in mitigating facial BCCs in Gorlin/NBCCS patients without causing systemic adverse effects commonly seen in oral Hh inhibitors [187]. A phase III confirmatory clinical trial study following this has just been recently completed in 2021, but no results have been posted so far (NCT03703310).

Like BCCs, most Shh-medulloblastomas are characterized by mutations in the *PTCH1* gene, and to a smaller extent, in *SUFU* and *SMO* genes [196]. Likewise, Shh-medulloblastomas are also amenable to SMO inhibitor treatment; in particular, sonidegib has shown superior antitumor efficacy (ORR: 55%) compared to vismodegib (ORR: 17%) in a systemic review and meta-analysis of phase I and phase II clinical trials of pediatric and adult medulloblastoma patients treated with sonidegib and vismodegib [197]. Currently, a phase I clinical trial evaluating the safety and tolerability of sonidegib in combination with ribociclib (NCT03434262) and a phase II clinical trial evaluating the feasibility and tolerability of vismodegib as maintenance therapy after conventional adjuvant chemotherapy (NCT01878617) are ongoing.

For other cancer types where mutations in Hh pathway genes are mostly absent, the utility of SMO inhibitors as monotherapy is less profound. Notably, glasdegib, in combination with low-dose cytarabine (LDAC), has been FDA approved to treat newly diagnosed AML patients who are 75 years old or older or have comorbidities that preclude the use of intensive induction chemotherapy. Approval was based on a multicenter, open-label, phase II study, which showed almost two-fold improvement in overall survival (OS) for the glasdegib + LDAC compared to LDAC alone arm [183,184]. A phase III clinical trial study is currently ongoing for the testing of saridegib in combination with intensive (glasdegib plus cytarabine and daunorubicin) or nonintensive (glasdegib plus azacitidine) chemotherapy (BRIGHT AML 1019; NCT03416179). Other hematological malignancies, such as hypomethylating agent (HMA) failure myelodysplastic syndrome (MDS) and HMA-relapsed AML, have also shown stable disease (SD) that was significantly correlated to improved OS when treated with SMO inhibitors, suggesting that SMO inhibition may add to HMA efficacy [168,171]. Additionally, lenalidomide in combination with LDE225 as post-transplant maintenance therapy in multiple myeloma patients who underwent single autologous stem cell transplant was associated with improved response depth [169].

Besides hematological-related malignancies, solid tumors such as pancreatic, gastric, breast cancer, and lung cancer may benefit from treatment with specific SMO inhibitors. In advanced pancreatic cancer, adding saridegib/IPI-926 to FOLFIRINOX (5-fluorouracil, leucovorin, irinotecan, and oxaliplatin) resulted in favorable ORR (67%), improved disease stabilization, and a decline in CA19-9 tumor marker [198]. Similarly, treatment of metastatic pancreatic cancer with combined saridegib and gemcitabine showed 31% radiological partial response (PR) [186]. However, the addition of a different SMO inhibitor, vismodegib, to gemcitabine and nab-paclitaxel did not improve efficacy compared to gemcitabine alone in patients with metastatic pancreatic cancer [159].

The addition of vismodegib to FOLFOX neither improved progression-free survival (PFS), response rate (RR), nor OS in advanced gastric and gastroesophageal junction carcinoma [199]; however, a subset of gastric and gastroesophageal junction cancer patients with high CD44 median score experienced CR, better OS, and reduced disease progression [200]. By contrast, treatment of advanced and recurrent gastric cancer with combined itraconazole, S-1, oxaliplatin, and nab-paclitaxel showed a high tumor RR (70%) and durable 1-year OS rate (81.8%) [191].

The addition of sonidegib to docetaxel also showed antitumor activity at the recommended phase II dose (RP2D) in 3 out of 10 triple-negative breast cancer patients with measurable disease, including one CR and two long-lasting stabilization [179]. Eleven out of fourteen patients diagnosed with extensive-stage small-cell lung cancer (SCLC) develop PR when treated with sonidegib in combination with etoposide/cisplatin [201], which was higher than historical rates observed with etoposide/cisplatin alone [202,203] in extensive SCLC patients. Overall, these results should be interpreted with caution as these clinical studies are limited by small numbers and lack a placebo arm or correlative molecular analyses to assess the relationship between response and expression of Hh pathway targets. Further studies are warranted to properly denote the therapeutic significance of targeting Hh-GLI signaling in treating these cancers.

GLI proteins, especially GLI1, have demonstrated prognostic values for predicting survival among diverse cancer types, including breast cancer [204,205,206,207,208], liver cancer [208,209], pancreatic cancer [208,210], ovarian cancer [208,211,212,213], glioma [214], prostate cancer [215], colon/colorectal cancer [81,216,217] gastric cancer [218,219,220], AML [208], and medulloblastoma [221]. By contrast, despite the widespread use of SMO inhibitors, the value of SMO as a prognostic biomarker is less profound and has only been reported in a handful of cancer types, including bladder cancer [222], glioma [223,224], liver cancer [68], and head-and-neck cancer [209]. However, SMO mutants have shown to be significantly associated with shorter survival in malignant pleural mesothelioma [225] and olfactory groove meningioma [226] patients, suggesting that SMO mutational features defining a molecular subgroup in cancers are better indicators of poor prognosis than wild-type SMO. As GLI1 is most amenable to activation by both SMO-dependent and SMO-independent axes among the Hh pathway components, it is unsurprising that its expression is more commonly associated with poor prognosis in Hh-active cancer patients.

Although GLI1 expression has been frequently associated with poor prognosis in many known cancers, SMO inhibitors are ineffective in treating various cancers despite showing antitumor activity in preclinical studies. Indeed, most preclinical studies utilized in vitro cell lines that do not fully represent in vivo tumor biology and physiology occurring in humans. Although patient-derived xenograft models may provide more physiologically relevant results, they lack the complex interplay between tumor cells and the tumor microenvironment components, including CAFs, immune cells, soluble growth factors, extracellular matrix, and the vasculature system [227]. In actuality, a tumor bulk includes a heterogeneous pool of cells that harbor distinct molecular signatures with differential levels of treatment sensitivity.

With regards to the above, the bulk of the tumor might include a distinct group of cells that utilizes either an SMO-dependent or SMO-independent route of GLI activation, or both—with the SMO-independent one likely being the preferential route supported by the fact that mutations in Hh components upstream of GLI are largely absent in most cancer types [228]. Furthermore, the major oncogenic signaling pathways frequently activated in cancers are proven to regulate GLI transcription and activity independent of SMO (see Section 3.2.1 and Section 3.2.2), potentially explaining why SMO inhibitors may be ineffective in producing meaningful tumor responses in most solid tumors despite the expression of GLI proteins in these tumors. In further support of this, a large-scale pancancer analysis revealed that *GLI1* and *GLI2* shared broad prognostic association with *TGFB* ligand and mesenchymal genes but not with Hh genes, potentially explaining the frequent failure of SMO inhibitors in most solid tumors [142]. Thus, targeting GLI may be a better therapeutic option in circumstances where GLI-expressing tumors do not respond well to SMO inhibitors.

Consistent with its role as a prognostic marker, GLI1 expression is commonly used as a pharmacodynamic marker in clinical studies. Additionally, GLI1 serves as an excellent biomarker for tumor response in BCC and Hh-active medulloblastoma [187,229,230]. However, decreased GLI1 levels do not always correlate with tumor response. Several clinical studies showed that the downregulation of intratumoral GLI1 levels did not correlate with tumor response in pancreatic and prostate cancer patients treated with SMO inhibitors [157,158,180]. In HM failure MDS patients treated with glasdegib as monotherapy, improved disease stabilization and survival did not correlate with Hh pathway expression but did halt further increase in *GLI1* mRNA and its downstream targets, including *MYC* and *CCND1* [171]. In such circumstances, correlative analyses of potential biomarkers with tumor responses may help identify a subset of patients who may derive benefit from SMO inhibitors.

However, Hh pathway components, particularly GLI1, are ideal predictive biomarkers for SMO inhibitor therapy in several cancers, including BCC and Shh-medulloblastomas. For instance, patients with BCC that responded well to sonidegib and vismodegib had upregulated *GLI1* transcripts [194,230]. In patients with Hh-positive medulloblastomas, five out of ten patients showed an objective response (OR), including four CRs and one PR, associated with elevated *GLI1* signature [174]. Patients with stable disease of 6 months or longer had grade 1 or 2 conventional chondrosarcomas, all of which showed overexpression of the Hh ligand [231]. An improved clinical response, including one CR, was also observed in three out of ten evaluable triple-negative advanced breast cancer patients with high paracrine Hh Pathway Activation Signature (HPAS), characterized by high tumor epithelial Hh and stromal GLI1 expression [179]. Thus, stratification of patients based on Hh biomarkers, particularly GLI1, may help identify a specific subset of patients that may benefit from Hh inhibitors.

## 5. Current Challenges and Future Perspective for Using SMO/GLI Inhibitors in Clinical Settings

SMO inhibitors have shown promising efficacy in treating Hh-active cancers, particularly BCC and Shh-medulloblastoma. Despite this, *SMO* mutations frequently emerged among these cancers, contributing to the development of acquired resistance against SMO inhibitors. The majority of acquired resistance is caused by mutations in the drug-binding pocket of SMO, impeding the binding of SMO inhibitors [63,64,67]. In light of this discovery, efforts are being made to develop nonredundant SMO inhibitors, such as TAK441 [232], taladegib [233], and LEQ-506 [234]. These investigational SMO inhibitors have been shown to inhibit SMO D473H mutant conferring resistance to vismodegib/sonidegib in preclinical studies [235]. Importantly, taladegib treatment has shown significant antitumor activity in Hh treatment-naïve and previously Hh-treated BCC patients of a phase I study [190], warranting further study on its utility as second-line therapy for treating SMO inhibitor-resistant cancers in clinical settings. The screening of benzimidazole derivatives led to the identification of novel SMO inhibitors, HH-13 and HH-20, with potent inhibitory activity on SMO D473H mutant conferring resistance to vismodegib [236]. Another novel SMO inhibitor, MRT-92, was shown to bind effectively to the entire transmembrane cavity of the SMO D473H mutant, which allows for the inhibition of the SMO mutant [237]. With regards to what is above, further study and characterization of these SMO inhibitors are still required to determine their suitability for proof-of-concept in vivo testing before they can be further tested in clinical settings.

Besides nonredundant SMO inhibitors, targeting GLI may serve as a feasible approach to overcoming SMO resistance. The FDA-approved antipinworm agent, pyrvinium, has been shown to inhibit the activity of SMO-D473H mutant and GLI activity resulting from the loss of SUFU [238]. Targeting the GLI protein function with arsenic trioxide or PSI was shown to circumvent the issue concerning SMO resistance in MEF cells, underscoring the use of GLI inhibitors as a second line of therapy [64]. Indeed, in a phase II clinical trial, the sequential treatment of metastatic BCC patients experiencing relapse after SMO inhibitor treatment with arsenic trioxide and itraconazole effectively suppressed 75% *GLI1* mRNA expression and produced SD in three out of five patients; however, the lack of tumor shrinkage may be due to suboptimal dosing or transient *GLI1* suppression [153]. Moreover, targeting GLI can also serve as a promising therapeutic approach for treating cancers with intrinsic resistance to SMO inhibitors due to *SUFU* mutations or *GLI2* amplification. Furthermore, oncogenic mutations in *GLI* genes have rarely been reported in cancers [239], making them a more predictable target for inhibition, but whether long-term usage of GLI inhibitors may lead to clinically acquired resistance remains a question.

Many solid tumors that have shown promise for the targeting of SMO in preclinical studies fail to translate successfully into clinical settings. Furthermore, the inferior prognostic and predictive value of SMO compared to GLI (see Section 4), the absence of *SMO* mutations despite the high expression of GLI [228], and the frequent report of a noncanonical arm of GLI activation in most cancers (see Section 3.2.1 and Section 3.2.2) strongly suggest an SMO-independent route as the major mechanism of GLI regulation in the majority of tumors. Indeed, various preclinical reports have shown a lack of *GLI* suppression and antitumor efficacy following treatment with SMO inhibitors that can be circumvented with GLI inhibitors (see Section 3.2.1 and Section 3.2.2). Furthermore, GLI inhibitors have shown superior pharmacological properties compared to SMO inhibitors [15,16].

Although preclinical results are convincing, most GLI inhibitors have yet to be assessed in the clinical setting due to their nonstable pharmacokinetics/bioavailabilities. For instance, GANT61 is rapidly hydrolyzed into an inactive benzaldehyde species due to its lack of stability at physiological pH [240]. Ongoing efforts are being made to develop more effective and stable GLI inhibitors for their use in clinical settings. Recently, a novel GLI inhibitor, SRI-38832, has shown not only an improved efficacy but also an enhanced bioavailability in a murine xenograft model, representing the first step toward the development of a clinically viable GLI inhibitor [241]. The improved efficacy and stable pharmacokinetic/bioavailability profiles of SRI-38832 make it a novel scaffold for hit-to-lead optimization, which, if successful, sets a new stage for treating Hh-dependent cancers in future clinical settings. Other potential GLI inhibitors include small chemical molecules, HPI-1 [242,243], HPI-2, HPI-3, and HPI-4 [242], and natural compounds, cynanbungeigenin C (CBC) and D (CBD) [244], physalin B [245], and glabrescione B [246]. An indirect approach of inhibiting GLI function by targeting GLI modulators has also been reported with bromodomain-4 (BRD4), CK2, aPKC, dual-specificity tyrosine phosphorylation-regulated kinase 1B (DYRK1B), and HDACs inhibitors [244,247].

Of note, the lack of efficacy with SMO inhibitors in treating solid tumors does not necessarily preclude its clinical significance in these tumors. When used in combination with chemotherapeutics, SMO inhibitors have shown antitumor activity in a specific subset of populations expressing CSC markers. For instance, in a small subset of patients with gastric cancer expressing high median CD44 scores, the addition of vismodegib to FOLFOX led to a better therapeutic response, including CR, better OS, and reduced disease progression. Furthermore, high CD44 expression was associated with decreased survival in the chemotherapy alone group. The authors’ preclinical work revealed that the CD44-expressing subpopulation of gastric cancer cells exhibited enhanced chemotherapeutic resistance, which can be reversed in vitro and in xenografts with SMO shRNA or vismodegib treatment [199].

Triple-negative breast cancer patients with elevated phospho-FGFR expression, collagen deposition, and fiber linearization experienced CR or disease stabilization when treated with combined sonidegib and docetaxel. On the other hand, nonresponders exhibited weak phospho-FGFR expression, low collagen deposition, and minimal evidence of mechano-signaling and breast CSCs despite having high paracrine HPAS. The authors demonstrated that Hh ligand secreted from murine triple-negative breast cancer cells promoted the reprogramming of CAFs, which led to the extensive reshaping of ECM that fostered the development of supportive CSC niches through enhancing mechano-signaling and phosphor-FGFR activation in the tumor epithelium [58].

A patient with extensive SCLC showing high tumor-specific amplification of *SOX2* and *PIK3CA*, both on chromosome 3q26.3, had the longest progression-free response for up to 27 months with maintenance sonidegib after several cycles of combined treatment with etoposide/cisplatin and sonidegib [200]. Notably, coamplification of *SOX2* and *PIK3CA* on chromosome 3q26 has been shown to cooperatively drive stem-like phenotype in tumor-derived lung squamous cell carcinoma cells by activating cell-autonomous Hh signaling axis, while treatment of these cells but not their parental counterpart with sonidegib significantly inhibited oncosphere proliferation [248]. These results support the feasibility of combining chemotherapeutics and SMO inhibitors for treating tumors, based on the notion that chemotherapeutics perform the killing of most fast-growing cancer cells, while SMO inhibitors target residual CSCs with active Hh signaling to halt tumor self-renewal and repopulation. Thus, the stratification of patients based on predictive biomarkers, such as Hh and possibly stem cell factors, may assist the identification of patients eligible for Hh-based therapies.

Despite the success of oral SMO inhibitors, their clinical use is restricted due to adverse effects frequently associated with these drugs. Adverse effects accompanied by vismodegib treatment frequently led to the discontinuation of treatment in patients with BCC, following which tumor recur [249]. In the open-label STEVIE trial, adverse effects were reported in 36% of advanced BCC patients treated with vismodegib, of which 22% were serious adverse effects [164]. The combined use of IPI-926 and FOLFIRINOX produced a high ORR rate of 67% in advanced PDAC patients, but detrimental effects induced by this combination in a phase II trial led to the unfortunate early termination of the study [197]. The development of a new topical Hh inhibitor, patidegib, has led to significant shrinkage of surgically eligible BCC lesions without any of the adverse effects usually seen in oral Hh inhibitors [187]; a phase III clinical trial has been conducted to confirm these findings. However, such inhibitors are only applicable to superficial cancer and do not circumvent the in vivo toxicity posed by most oral Hh inhibitors when treating nonsuperficial or invasive cancers.

Ongoing efforts are still being made to discover novel potent SMO inhibitors, such as MRT-83, MRT-92, CAT3, MK-4101, Smoothib, L-4, Nilotinib [244], and naturally derived compounds [245]. Importantly, discovering new hit compounds can provide a platform for the innovative design of new derivatives that are potentially safer yet still effective for treating SMO-dependent cancers in clinical settings. Of importance, the use of GLI inhibitors may produce less-adverse effects compared to SMO inhibitors due to their improved pharmacological properties, but intensive efforts are still needed to develop more pharmacologically stable GLI inhibitors before they can be further tested in clinical trials.

It has been recognized that combination treatment may allow for synergistic interaction that allows the administration of lower doses of the combination constituents, thereby reducing adverse reactions. With that said, simultaneous targeting of SMO and GLI has been shown to provide synergistic inhibitory effects on various cancers, including multiple myeloma [250,251], medulloblastoma [252], and glioblastoma [253]. Additionally, a combination of SMO inhibitors with inhibitors targeting other oncogenic targets, such as PI3K/AKT/mTOR [254,255,256,257], EGFR [258], DNA methyltransferases [83], and interleukin 6 [259], resulted in synergistic inhibition of cancer cell growths. Such combinations included the use of SMO or GLI inhibitors with drugs targeting oncogenic drivers of noncanonical GLI activation, which allows for the simultaneous targeting of compensatory noncanonical GLI activation and other Hh-unrelated key cancer targets.

Recently, glasdegib added to LDAC has demonstrated improved clinical efficacy for treating AML compared to SMO inhibitors given as monotherapy [156,172,183,184]; such findings were also demonstrated in preclinical studies [151]. Additionally, vismodegib plus arsenic trioxide, in association with temozolomide, resulted in the marked inhibition of glioblastoma tumor growth in mice, while single-agent therapy yielded minimal efficacy [253]. Cotreatment of HT29 cells with GANT61 and 5-fluorouracil produced a synergistic and marked inhibitory effect compared to treatment with any of the agents alone [241].

Currently, ongoing efforts are being made in clinical trials to investigate the suitability of SMO inhibitors as part of a combination therapy regimen for treating various cancers (see Table 2 and Section 4). Taken together, the current preclinical and clinical data support the potential for synergistic effect when the SMO inhibitor is administered alongside conventional chemotherapeutics or other targeted drugs, representing a plausible approach for reducing adverse reactions while providing optimal clinical responses. Such an approach should be further considered for tumors such as mBCC and Hh-active medulloblastoma that have shown high tumor response to Hh inhibitors, which may improve the odds of therapeutic success. Furthermore, the constant emergence of SMO inhibitor-resistant tumors in monotherapy treatment points further to the need to investigate the use of SMO inhibitors as part of a combination therapy regimen.

## 6. Conclusions

This review highlighted the diverse biological roles of GLI transcriptional effectors in cancer initiation and progression. GLI proteins can be regulated through SMO-dependent and SMO-independent mechanisms, both of which have been heavily implicated in tumorigenesis. SMO-dependent GLI signaling occurs as a result of dysregulated upstream Hh components caused by mutations (e.g., loss-of-function of PTCH and gain-of-function of SMO) or uncontrolled transcriptional regulation (e.g., aberrant transcription factors and epigenetic alterations), resulting in the hyperactivation of SMO by which the excessive signal is translated to GLI. Conversely, SMO-independent GLI signaling involves the noncanonical crosstalk of GLI with other signaling pathways (KRAS/MAPK/ERK, TGF-β/SMAD, TNF/PI3K/AKT/mTOR, Wnt/β-catenin, and NF—kB) and signaling proteins. This method of GLI regulation is commonly implicated in cancers that are resistant to SMO inhibitors (e.g., cyclopamine and vismodegib).

SMO inhibitors have proven to be effective in treating a wide range of malignancies in clinical settings, especially in cancers such as BCC that are heavily dependent on Hh-GLI signaling. However, resistance to SMO inhibitors often arises due to mutations within the LBPs of SMO, consequently blocking the binding of inhibitors. Moreover, the noncanonical route of GLI activation has been frequently reported in many other solid tumors, such as NSCLC, PDAC, gastric adenocarcinomas, and triple-negative breast cancer, leading to SMO inhibitor resistance. Hence, we propose that targeting GLI transcription factors shows promise as a therapeutic strategy for overcoming SMO-independent cancers’ growth. Firstly, oncogenic mutations are less commonly found in GLI proteins as compared to other Hh pathway components, making them a more fitting therapeutic target that is less likely to develop resistance. Secondly, inhibiting GLI protein function has proven to exert superior anticancer effects compared to targeting Hh components upstream of GLI proteins. Thirdly, in many Hh pathway overexpressing cancers, GLI proteins (especially GLI1) are the most consistently upregulated component among the Hh pathway elements and are intimately associated with poor prognosis in cancer patients, making them more practical therapeutic targets in a wide variety of tumors. Lastly, inhibiting the GLI function serves as a promising strategy for blocking both canonical and noncanonical input and may serve as an alternative therapeutic method for treating SMO inhibitor-resistant cancers.

Future efforts should focus on optimizing potential lead Hh-targeting compounds to improve their potency, stability, solubility, pharmacokinetic profiles, specificity, and safety. One such compound, GLI inhibitor SRI-38832, has shown promise as a scaffold for hit-to-lead optimization, as shown by its stable pharmacokinetic/bioavailability profiles and good efficacy in the xenograft murine model, representing the first step toward the development of a clinically viable GLI inhibitor. An investigational SMO inhibitor, taladegib, has demonstrated a potent inhibitory effect on SMO D473H mutant and showed promising antitumor efficacy in Hh treatment-naïve and previously Hh-treated BCC patients, showing promise as second-line therapy for SMO-inhibitor resistant cancers. Efforts can be made to identify and validate new predictive biomarkers to help select potentially responsive patients who may benefit from Hh therapies. Lastly, including Hh inhibitors as part of a combination therapy regimen may help improve clinical efficacy while minimizing the emergence of acquired resistance and reducing adverse reactions.

## Figures and Tables

**Table 1:** Summary of the described SMO-dependent and SMO-independent mechanisms of GLI activation in cancers and the cancer hallmarks involved.

GLI Activation	Dysregulation	Regulators	Mechanism of Action	Cancer/Cell Type	Cancer Hallmarks	References
SMO-dependent	Mutations	PTCH1	Inactivating PTCH1 mutation leads to SMO derepression and GLI1/2 activation	Basal cell carcinoma	Proliferation, resisting cell death, angiogenesis, genomic instability, invasion, metastasis, evading growth suppressor	[44,45,49,50,51,52]
				Medulloblastoma	Proliferation	[53,54]
				Odontogenic keratocystic tumors	Proliferation, tumor-promoting inflammation	[55]
				T-cell acute lymphoblastic leukemia	Proliferation, resisting cell death	[56]
				Breast cancer	Stemness, resisting cell death	[57,58]
				Cervical carcinoma	Resisting cell death, invasion and metastasis	[59,60,61]
		SMO	SMO mutants constitutively activate GLI1/2 in the presence of vismodegib and are resistant to PTCH catalytic inhibition	Basal cell carcinoma	Proliferation, resisting cell death, angiogenesis, genomic instability, invasion and metastasis, and evading growth suppressor	[44,49,50,51,52,62,63,64,65,66]
			SMO mutant constitutively activate GLI1 in the presence of vismodegib	Medulloblastoma	Resisting cell death	[67]
			SMO mutant leads to enhance GLI1 expression and is more resistant to cyclopamine	Hepatocellular carcinoma	Proliferation	[68]
	Transcriptional	NFκB	Transcriptionally upregulates Shh at the promoter level, leading to canonical Shh-GLI activation	Pancreatic cancer	Proliferation, resisting cell death, tumor-promoting inflammation	[69,70,71,72,73]
				Breast cancer	Stemness, activating migration	[74,75]
		CREB, AP1, AP2α, and SP1	Transcriptionally upregulates SMO at the promoter level, leading to GLI activation	Prostate and breast cancer	NS	[76]
		β-catenin/TCF-4	Transcriptionally upregulates both SMO and GLI at the promoter level	Foreskin fibroblast	Proliferation	[77]
	Epigenetic	DNA methyltransferase	Hypomethylation of Shh promoter leads to improve NFκB-induced Shh transcription and GLI1 expression	Breast cancer	Stemness, migration	[74]
			Hypomethylation of SMO promoter leads to improve SMO transcription and subsequent GLI3 activation	Colorectal cancer	Stemness, proliferation, invasion, deregulated cellular energetic	[78,79,80,81,82]
			Hypomethylation of SMO promoter leads to improve SMO transcription and subsequent GLI2 expression	prostate, kidney, glioblastoma, and ovarian cancer	NS	[76]
			Hypermethylation of PTCH1 promoter leads to decrease PTCH1 expression, causing enhance SMO-GLI1/2 activation and GLI1/2 nuclear translocation	Leiomyosarcoma	Proliferation, activating migration, resisting dell death	[83]
			Hypermethylation of PTCH1 and HHIP lead to increase Hh-GLI signaling	Gastric cancer	Resisting cell death, proliferation, invasion and metastasis	[84,85,86,87,88,89,90]
SMO-independent	Oncogenic pathways	MAPK/ERK	Stimulation of NRP2 by VEGFa activates ERK, which phosphorylates GLI1 to promote its activation	Lung adenocarcinoma	Stemness, resisting cell death, angiogenesis	[91,92]
			Stimulation of NRP2 by VEGF induced α6β1 integrin-mediated activation of RAS/MEK signaling through focal adhesion kinase FAK activation and consequently GLI1 expression	Breast cancer	Stemness, resisting cell death	[58,93]
			Oncogenic mutant KRAS enhances GLI1 expression via RAF-MEK1-ERK	Pancreatic ductal adenocarcinoma	Resisting cell death, proliferation, invasion and metastasis	[94,95]
		TGF-β/SMAD	TGF-β enhances GLI1 expression	Hepatocellular carcinoma	Stemness, proliferation, migration, and invasion	[96]
				Pancreatic ductal adenocarcinoma	Resisting cell death, proliferation	[94]
			TGF-β/SMAD3 enhances GLI1 and GLI2 expression	Melanoma	Resisting apoptosis, proliferation, and invasion	[97]
		Cooperative integration of TGF-β/SMAD and Wnt/β–catenin	β–catenin/TCF-4 and SMAD cooperatively bind to the GLI2 promoter and enhance its transcription	Breast cancer	Invasion and metastasis	[98]
				Oral squamous cell carcinoma		[99]
		Wnt/β–catenin	β–catenin/TCF-4 upregulates CRD-BP, which binds and stabilizes GLI1 transcripts	Colorectal cancer	Proliferation	[100,101]
		PI3K/AKT	p-AKT enhances GLI1 expression	Gastric cancer	Proliferation, migration, invasion, metastasis, resisting cell death, avoiding immune destruction	[102,103,104]
			PI3K/mTOR regulates GLI1 expression	Lung squamous cell carcinoma	Proliferation	[105]
			ErbB2 enhances GLI1 expression via PI3K/AKT/mTOR activation	Esophageal adenocarcinoma	Proliferation, resisting cell death	[106]
			PI3K/AKT regulates the nuclear translocation of GLI1	Osteosarcoma	Resisting cell death	[107]
			DYRK1B activates PI3K/AKT/mTOR pathway to promote GLI1 stabilization	Pancreatic and ovarian cancer	Proliferation	[108]
			PI3K/AKT enhances GLI1/2 expression and nuclear translocation	Renal cell carcinoma	Proliferation, resisting cell death	[109]
			TNFα induces S6K1 phosphorylation and GLI1 expression	Prostate cancer	Proliferation	[110]
			TNFα/mTOR activation of S6K1 induces phosphorylation of GLI1, promoting its stability	Esophageal adenocarcinoma	Proliferation and invasion	[111]
			p70S6K2 phosphorylates and inhibits GSK3β function, promoting GLI1 stability	Non-small cell lung cancer	Proliferation, resisting cell death	[112]
		RAS-MEK/AKT	Endogenous RAS-MEK and AKT signaling regulate GLI1 transcription and nuclear localization	Melanoma	Proliferation, resisting cell death, metastasis	[113]
		NFκB	P65 transcriptionally upregulates GLI1 expression by binding to GLI1 promoter	Breast cancer	Cell proliferation, stemness, migration	[114]
	Oncogenic proteins	SOX-9	SOX9 binds and inhibits β-TrCP, promoting GLI1 stability	Pancreatic ducal adenocarcinoma	Stemness, proliferation, and resisting cell death	[115]
		FOXC1	FOXC1 binds to GLI2 and enhance its DNA binding	Breast cancer	Stemness, proliferation, resisting cell death	[116]
		Nestin	Nestin binds to GLI3 to prevent phosphorylation by PKA, thereby enhancing GLI3 stability	Medulloblastoma	Proliferation	[117]
		Gal-1	Gal-1 enhance GLI1 expression by binding and activating β1 integrin	Gastric cancer	Invasion, metastasis, vasculogenic mimicry, avoiding immune destruction	[118,119,120,121]
			Gal-1 enhances GLI1 expression	Pancreatic ductal adenocarcinoma	Proliferation, angiogenesis	[122]
	Tumor suppressors	SPOP	Downregulation of SPOP enhance GLI1/2 expression	Ovarian cancer	Proliferation, resisting cell death	[123]
			Downregulation of SPOP prevents proteasomal-dependent degradation of GLI2, thus promoting its stability	Gastric cancer	Proliferation, migration, and resisting cell death	[124]
				C3H10T1/2	NS	[125]
		ASPP2	Downregulation of ASPP2 enhances aPKC-ι-mediated phosphorylation of GLI1 to promote its nuclear translocation	Gallbladder cancer	Invasion, metastasis, and tumor-promoting inflammation	[126]
		GSK3β	Deregulated GSK3β function as a result of imbalance activating and inactivating phosphorylation impaired its ability to phosphorylate GLI3, promoting GLI3 stabilization	Colon cancer	Proliferation and resisting cell death	[127]
		MED12	Downregulation of MED12 relieve its constraint on GLI3, promoting its hyperactivation	Prostate cancer	Proliferation	[128]

AKT, protein kinase B; ASPP2, apoptosis-stimulating of p53 protein 2; CRD-BP, coding region determinant binding protein; DYRK1B. dual-specificity tyrosine phosphorylation-regulated kinase 1B; ErbB2, erb-b2 receptor tyrosine kinase 2; ERK, extracellular-signal-regulated kinase; FAK, focal adhesion kinase; FOXC1, transcription factor forkhead box C1; Gal-1, galectin-1; GLI1/2/3, glioma-associated oncogene homolog 1/2/3; GSK3β, glycogen synthase kinase 3 beta; HHIP, hedgehog interacting protein; KRAS, kirsten rat sarcoma 2 viral oncogene homolog; MAPK, mitogen-activated protein kinase; MED12, mediator of RNA polymerase II transcription subunit 12; mTOR, mechanistic target of rapamycin kinase; MEK, mitogen-activated protein kinase kinase; NFκB, nuclear factor kappa B; NRP2, neuropilin 2; NS, not specified; PI3K; phosphoinositide 3-kinase; PKA, protein kinase A; PTCH1, patched 1; SMO, smoothened; SPOP, speckle-type POZ protein; TCF-4, T-cell factor 4; TGF-β, transforming growth factor-β; TNFα, tumor necrosis factor-alpha; and VEGFa, vascular endothelial growth factor A.

**Table 2:** Summary of HH inhibitors’ latest clinical findings in developmental clinical trials from 2017–2021.

Hh Inhibitor	Clinical Trial Phase	Cancer Type (Patients Enrolled)	Treatment Interventions	Efficacy	Clinical Trial Number (Recruitment Status)
Vismodegib	Phase Ib [155]	Intermediate or highrisk MF (*n* = 10)	Vismodegib 150 mg daily with ruxolitinib 15 or 20 mg twice daily	Only symptom response (*n* = 5)	NCT02593760 (Completed)
	Phase Ib [156]	Rel/ref AML (*n* = 38)	Vismodegib 150 mg once daily	ORR 6.1%	NCT01880437 (Terminated)
	Phase I [157]	Metastatic pancreatic cancer (*n* = 69)	Erlotinib 150 mg and vismodegib 150 mg once daily	No tumor response observed; SD (n = 13); paired biopsies analysis showed reduced GLI1 mRNA, phospho-GLI, and Hh target genes;	NS
	Phase I [158]	Pancreatic cancer or other solid tumors (*n* = 31)	Vismodegib 150 mg daily plus sirolimus at an increasing dose from 3 to 6 mg daily	SD (*n* = 6); No PR or CR observed, reduced GLI1 expression before and after the first cycle	NCT01537107 (Completed)
	Phase II [159]	Untreated PDA (*n* = 71)	Gemcitabine 1000 mg/m^2^ and nab-paclitaxel 125 mg/m^2^ × days 1-8-15, followed by the same regimen with oral vismodegib 150 mg daily × 28 days	ORR 27%, median OS 9.79 months, and median PFS 5.42 months	NCT01088815 (Completed)
	Phase II [160]	Multiple BCC (*n* = 229)	Group A: vismodegib 150 mg daily x 12 weeks, then placebo × 8 weeks, followed by vismodegib 150 mg daily × 12 weeks; Group B: vismodegib 150 mg daily x 24 weeks, then placebo × 8 weeks, followed by vismodegib 150 mg daily × 8 weeks	ORR 62.7% (Group A) and 54.0% (Group B)	NCT01815840 (Completed)
	Phase II [161]	laBCC (*n* = 55)	Vismodegib 150 mg once daily	CR 61.4%	NCT02667574 (Ongoing)
	Phase I [162]	laBCC (*n* = 71) and mBCC (*n* = 33)	Vismodegib 150 mg once daily until disease progression, intolerable toxicity, or study withdrawal	ORR 60.3% (laBCC) and 48.5% (mBCC); median OS 33.4 months (mBCC), not estimable in laBCC cohort	NCT00833417 (Completed)
	Phase II [163]	Infiltrative, Nodular, and Superficial BCC (*n* = 27)	Vismodegib 150 mg daily	CR 20%, PR 41.5%, and SD 36.9%	NCT01700049 (Completed)
	Phase II [164,165,166,167]	Italian cohort: laBCC (n = 159) and mBCC (*n* = 23)	Vismodegib 150 mg daily until progressive disease, unacceptable toxicity, or withdrawal	ORR 61.7% (laBCC) and 20% (mBCC)	NCT01367665 (Completed)
		Ocular or Periocular laBCC (*n* = 244)		CR 28.7% and PR 38.5%	
		laBCC and mBCC (*n* = 1232)		ORR 68.5% (laBCC) and 36.9% (mBCC); SD 25.1% (laBCC) and 46.4% (mBCC)	
		laBCC and mBCC (*n* = 1227)		ORR 64.9%	
Sonidegib/Erismodegib	Phase I [168]	Untreated AML (*n* = 15), rel/ref AML (*n* = 23), MDS (*n* = 18), CMML (*n* = 4), and MF (*n* = 2)	Sonidegib 200–400 mg daily with azacitidine 75 mg/m^2^	AML: ORR 23.1%; rel/ref AML: ORR 7.1%, SD 76%, and OS 7.6 months	NCT02129101 (Completed)
	Phase II [169]	Multiple myeloma (*n* = 28)	Sonidegib 400 mg daily with Lenalidomide 10 mg daily	CR 46%, VGPR 85%, and 24 month PFS 73%	NCT02086552 (Ongoing)
	Phase Ib/II [170]	MF without prior therapy with JAKi (*n* = 50)	Sonidegib 400 mg daily with ruxolitinib 20 mg twice daily	29.6% patients achieved > 35% reduction in spleen volume; 26% patients achieved 50% reduction in MFSAF and TSS; minimal change in GLI1 expression	NCT01787552 (Completed)
	Phase II [171]	Hypomethylating agent failure: MDS (*n* = 26), CMML (*n* = 5), and AML (*n* = 4)	Oral glasdegib 100 mg daily	ORR 6%, SD 56%, median OS 10.4 months, and EFS 6.4 months	NCT01842646 (Completed)
	Phase I [172]	AML (*n* = 7), MDS (*n* = 4), CMML (*n* = 1), and MF (*n* = 1)	Glasdegib 25/50/100 mg once daily	>80% suppression of GLI1 expression; AML: CR 8% and SD 31%; MDS: CR 8% and SD 16%	NCT02038777 (Ongoing)
	Phase Ib [173]	CML-CP (*n* = 11)	Erismodegib 200 mg once daily with nilotinib 400 mg twice daily	No clear clinical benefits were observed in terms of MMR and CCyR	NCT01456676 (Completed)
	I/II [174]	MB (*n* = 55) and others (*n* = 21)	Sonidegib 800 mg (adult) or 680 mg/m^2^ (pediatric) once daily	ORR 6.58% (50% responses were in Hh-positive MB patients); SD (*n* = 11; 27.7% responses were in Hh-positive MB patients)	NCT01125800 (Completed)
	Phase II [175]	mBCC (*n* = 36), laBCC: aggressive (*n* = 112) and nonaggressive (*n* = 82)	Sonidegib 200 or 800 mg once daily	ORR 51.1% (laBCC) and 12.6% (mBCC)	NCT01327053 (Completed)
	Phase II [176]	NBCCS (*n* = 10)	Sonidegib 400 mg or placebo	Total BCC reduced by 40% and 45% at weeks 12 and 16, respectively, vs. zero reduction for placebo	NCT01350115 (Completed)
	Phase Ib [177]	Metastatic pancreatic cancer: Chemo-naïve (*n* = 17) and prior-chemo (*n* = 9)	Escalated dose of sonidegib (800 mg and 200 mg for chemo-naïve and prior-chemo group, respectively) with gemcitabine 1000 mg/m^2^ and nab-paclitaxel 125 mg/m^2^	SD 8%, 35% PR, and 4% CR.	NCT02358161 (Completed)
	Phase I/II [178]	Metastatic pancreatic cancer (*n* = 25)	Sonidegib 200 mg once daily with gemcitabine 1000 mg/m^2^ and nab-paclitaxel 125 mg/m^2^	PR 10%, SD 53%, and OS 6 months	
	Phase Ib [179]	Triple-negative advanced breast cancer (*n* = 12)	Sonidegib 400/600/800 mg with docetaxel 75 mg/m^2^	ORR 30%	NCT02027376 (Completed)
	Phase I [180]	High-risk localized prostate cancer (*n* = 14)	Sonidegib 800 mg once daily or no treatment × 4 weeks before prostatectomy	86% in the Sonidegib arm achieved at least two-fold GLI1 suppression; no significant difference in DFS between sonidegib and observation arms	NCT02111187 (Completed)
Glasdegib	Phase Ib/II [181]	Primary or secondary MF treated previously with ruxolitinib (*n* = 21)	Glasdegib or placebo 100 mg once daily	9.5% and 40% patients had 50% and more than 20% reduced in TSS at week 12, respectively; SVR 4.8%	NCT02226172 (Terminated)
	Phase Ib [182]	AML or high risk MDS (*n* = 52)	Glasdegib 100 or 200 mg once daily, either with LDAC 20 mg twice daily, decitabine 20 mg/m^2^, or cytarabine 100 mg/m^2^ and daunorubicin 60 mg/m^2^	CR 31%	NCT01546038 (Completed)
	Phase II [183,184]	AML ineligible for intensive chemotherapy: de novo (*n* = 56) and secondary (*n* = 60)	Glasdegib 100 mg daily with LDAC 20 mg twice daily or LDAC 20 mg alone	Median OS: de novo (6.6 vs. 4.3 months) and secondary (9.1 vs. 4.1 months)	
		AML ineligible for intensive chemotherapy (*n* = 116)		Median OS 8.3 vs. 4.3 months	
	Phase II [185]	AML (*n* = 66) and MDS (*n* = 5)	Glasdegib 100 mg once daily with cytarabine 100 mg/m^2^ and daunorubicin 60 mg/m^2^	CR 46.4% (≥ 55 years old 40% CR), median OS 14.9 months	
Saridegib/Patadegib	Phase Ib [186]	Metastatic pancreatic cancer (*n* = 16)	Saridegib (110, 130, or 160 mg) once daily with gemcitabine 1000 mg/m2	Radiological PR 31% and median PFS > 7 month	NCT01130142 (Completed)
	Phase II [187]	Gorlin syndrome BCC (*n* = 17)	Vehicle or 2/4% patidegib twice daily	ORR 25% (patidegib) vs. 0% (vehicle); shrinkage of SEBs was observed only in patients with successful reduction in Hh pathway activity.	NCT02762084 (Completed)
Taladegib	Phase I [188]	Advanced solid tumors (*n* = 19)	Taladegib 100/200/300 mg once daily	All dose levels significantly inhibit GLI1 transcript levels; PR 5.3% and SD 21.1%	NCT01919398 (Completed)
	Phase I/Ib [189]	Advanced solid tumors (*n* = 16)	Taladegib 50/100 mg once or 400 mg twice daily with paclitaxel 80 mg/m^2^	PR (*n* = 3)	ISRCTN15903698 (NS)
	Phase I [190]	Treatment-naïve and previously treated BCC (*n* = 84)	Taladegib 400 mg once daily	Unaffected skin biopsies GLI1 expression showed an inhibition median of 92.3%; ORR 46.8%	NCT01226485 (Completed)
Itraconazole	Phase II [191]	stomach cancer and gastroesophageal junction cancer (*n* = 23)	160 mg/m^2^ nab-paclitaxel and 100 mg/m^2^ oxaliplatin x 1 day, S-1 60 mg/m^2^ × 3 days, and itraconazole 400 mg × days 3 days	ORR 70%, median OS 24 months, and 1-year OS rate 95%,	UMIN000021340 (Preinitiation)
	Phase II [192]	Biochemically recurrent prostate cancer (*n* = 21)	Oral itraconazole 300 mg twice daily	>50% PSA decline 5%; any PSA decline 47%; among 10 patients without a PSA decline, no significant difference between on-treatment and pretreatment PSADT	NCT01787331 (Completed)
	Phase I/II [193]	Platinum-resistant ovarian cancer (*n* = 11)	Itraconazole 300 mg twice daily with hydroxychloroquine (dose escalation 200–600 mg twice daily)	ORR none, SD (*n* = 1), and median PFS 1.6 months	NCT03081702 (Completed)
Arsenic trioxide	Phase II [153]	Refractory mBCC (*n* = 5)	Arsenic trioxide 0.3 mg/kg × 5 days followed by itraconazole 400 mg × 23 days	SD 60%; 75% reduction in GLI1 expression	NCT01791894 (Completed)

AML, acute myeloid leukemia; BCC, basal cell carcinoma; CCyR, complete cytogenic response; CML-CP, chronic myeloid leukemia in chronic phase; CMML, chronic myelomonocytic leukemia; CR, complete response; DFS, disease-free survival; EFS, event-free survival; GLI1; glioma-associated oncogene homolog 1; Hh, hedgehog; JAKi, januse kinase inhibitor; laBCC, locally advanced basal cell carcinoma; LDAC, low dose cytarabine; MB, medulloblastoma; mBCC, metastatic basal cell carcinoma; MDS, myelodysplastic syndrome; MF, myelofibrosis; MFSAF, myelofibrosis symptom assessment form; MMR, major molecular response; NBCCS, nevoid basal cell carcinoma syndrome; NS, not specified; ORR, overall response rate; OS, overall survival; PDA, pancreatic ductal adenocarcinoma; PFS, progression-free survival; PR, partial response; PSA, prostate-specific antigen; PSADT, prostate-specific antigen doubling time; rel/ref, relapsed or refractory; SD, stable disease; SEBs, surgically-eligible basal cell carcinomas; SVR, spleen volume reduction; TSS, total symptom score; and VGPR, very good partial response.
